# Supplementary material for: mTOR downregulation promotes anti-inflammatory responses via the CCL3-CCR5 axis in hypoxic retinopathy
Source: Mol Ther Methods Clin Dev. 2024 Dec 31;33(1):101404. doi: 10.1016/j.omtm.2024.101404 (PMC11787640; doi:10.1016/j.omtm.2024.101404)
Supplement: Document S1. Figures S1–S5, Table S1, and supplemental materials and methods [file mmc1.pdf]

OMTM, Volume *33*

Supplemental information

mTOR downregulation promotes anti-inflammatory responses via the CCL3-CCR5 axis in hypoxic retinopathy

Tae Kwon Moon, Im Kyeung Kang, Kyoung Jin Lee, Ji Hyun Kim, Hee Jong Kim, A. Reum Han, Ha-Na Woo, Joo Yong Lee, Jun-Sub Choi, Keerang Park, and Heuiran Lee

Table S1. Summary of AAV2-shmTOR-SD toxicological study in NHP

A. Test Facility: Korea Institute of Toxicology, KRICT

B. Animal: Cynomolgus Monkey (*Macaca fascicularis*), 30 (Male 15, Female 15) animals

C. Test tissue list

Tissue Sampled	
Eyes (cornea, sclera, choroid and retina, lens, vitreous humo r, optic nerve, lacrimal gland/sac/duct)	Cerebrospinal fluid (CSF, ≥0.5 mL)
Brain (3 sections: frontal, mid brain, cerebellum)	Liver
Spleen	Lungs
Kidneys (cortex and medulla)	Heart
Ovaries	Testes
Uterus with cervix	Epididymides
Skeletal muscle	Colon
Salivary glands (all potential routes of viral clearance)	Lymph nodes (submandibular)
Urinary bladder	Thyroid gland

D. Quality control and detection range by PCR

The analytical method is considered to be suitable to analyze AAV2-shmTOR-SD in Cynomolgus Monkey within the range of 4×10^2 to 4×10^7 copies/500 ng.

E. Summary of analysis results

Toxicology	The toxicity was observed for 1 3 weeks after single intravitreal injection in NHP (Cynomolgus macaque).	No test article-related adverse effects were observ ed in any parameter after a single intravitreal injec tion of AAV2-shmTOR-SD at doses of 2.5×10^9 o r 1.25×10^{10} vg/eye. Based on the results, the No Observed Adverse Effect Level (NOAEL) was co nsidered to be 1.25×10^{10} vg/eye in both male and female under this study condition.
T-cell response	IFN-γ ELISpot assays and TNF -α measurement were tested usi ng the PBMC samples of NHP bloods taken in toxicology stud y.<br>The sampling of PBMC were p erformed in 3 time points after administration.	No immune activation against AAV capsid protein was observed following treatment of AAV2-shmT OR-SD.<br>Increasing of TNF-α was only observed on day 3 after intravitreal injection. This response was not o bserved at other time points (Day 22 and day 92) a fter administration of AAV2-shmTOR-SD.
Biodistribution	The biodistribution of AAV2-sh mTOR-SD was observed in eye and 17 organs (or tissues) for 1 3 weeks.<br>The viral vector was measured by real-time PCR analysis.	AAV2-shmTOR-SD was distributed primarily in t he vitreous humor and around injection site of the injected eye after a single intravitreal injection in c ynomolgus monkeys. AAV2-shmTOR-SD was bel ow quantifiable levels in non-ocular tissues.

Figure S1

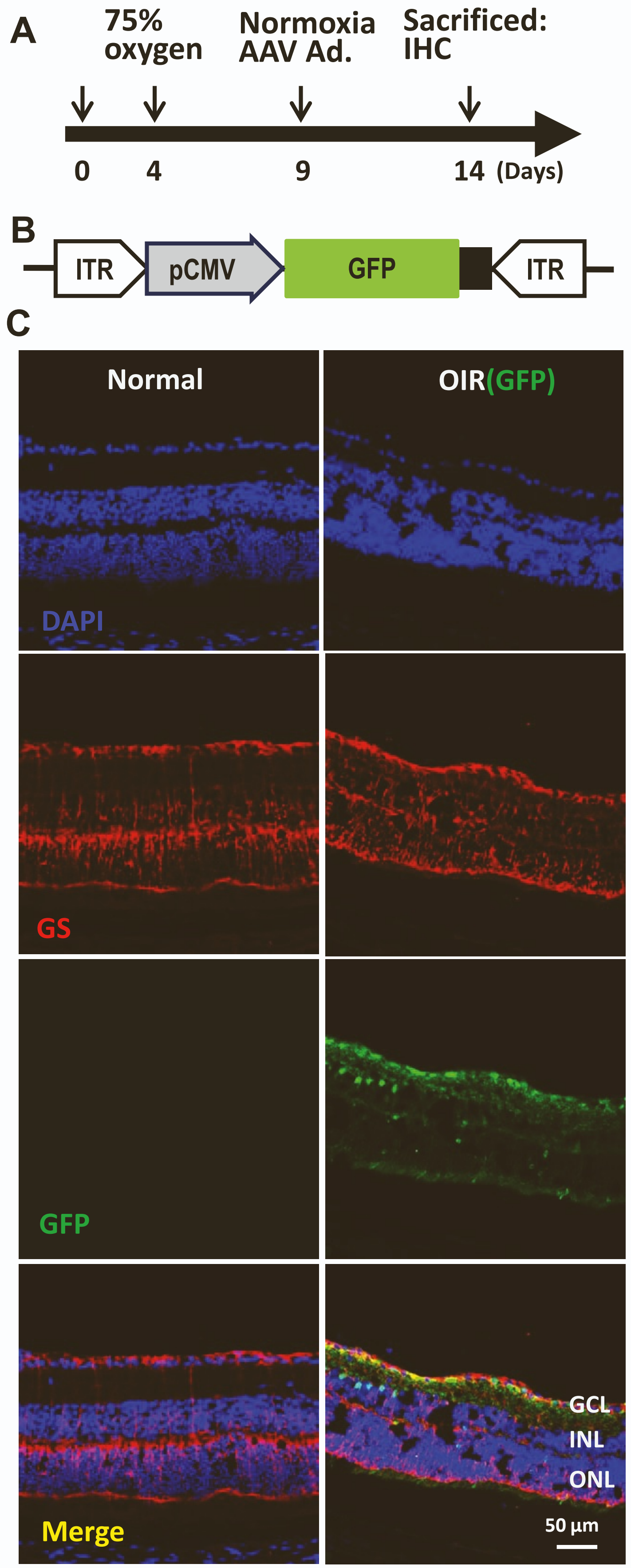

Figure S1. The transduction of **Müller cells by AAV2 in a rat OIR model.** (A) Diagram outlining the experimental setup. (B) Schematic overview of AAV2-GFP. (C) Immunohistochemistry targeting GFP and GS. The retina was analyzed after recovery to determine AAV2 tropism by immunohistochemically examining the GFP and GS expression pattern, demonstrating AAV's affinity for Müller cells. GCL, glial cell layer; INL, inner cell layer; ONL, outer cell layer.

Figure S2

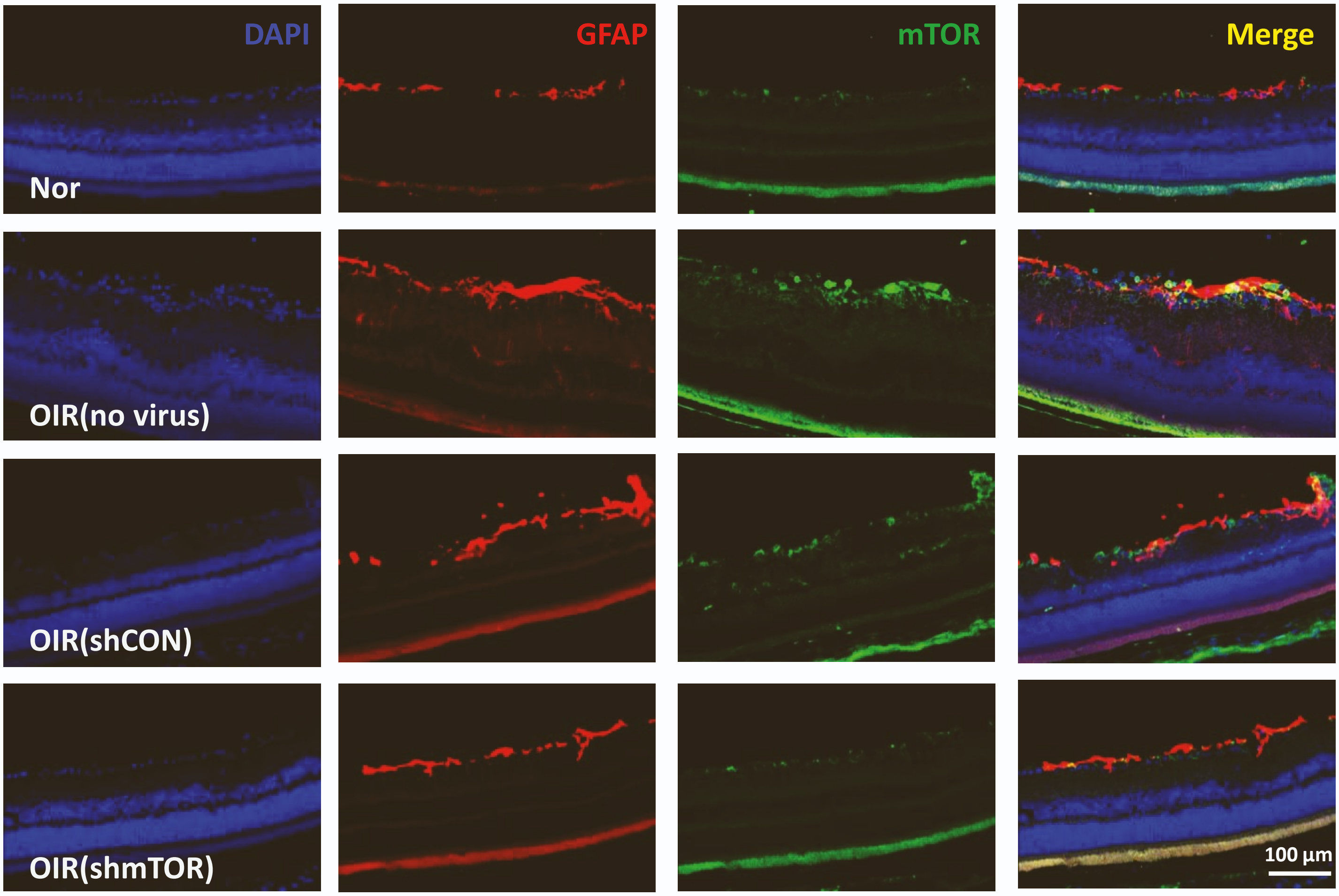

Figure S2. Immunohistochemical analysis targeting GFP and GFAP at low magnification of Fig. 1C.

Figure S3

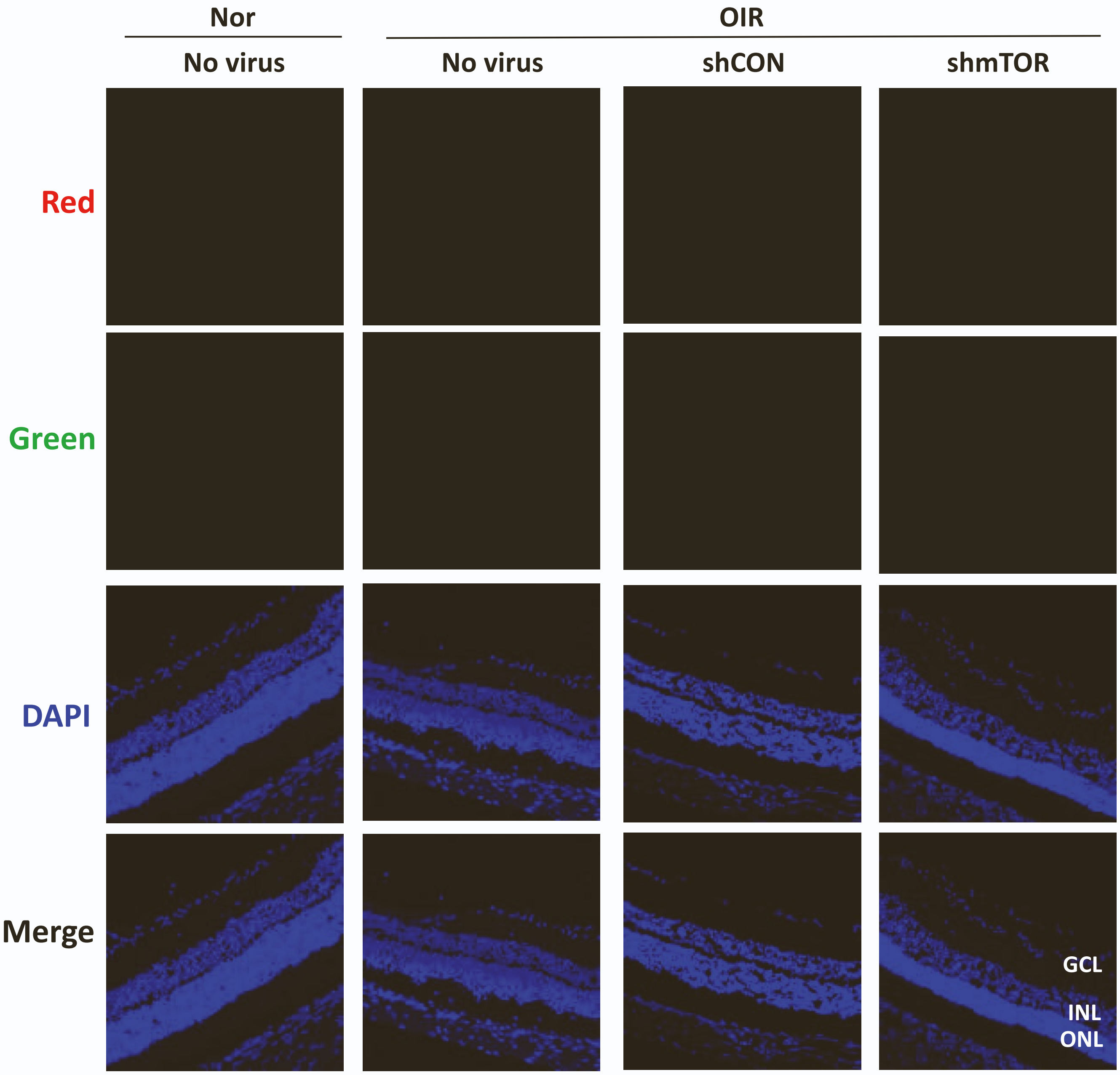

Figure S3. The immunohistochemical analysis was conducted without the use of a primary antibody, which served as a negative control of Fig. 1C.

Figure S4

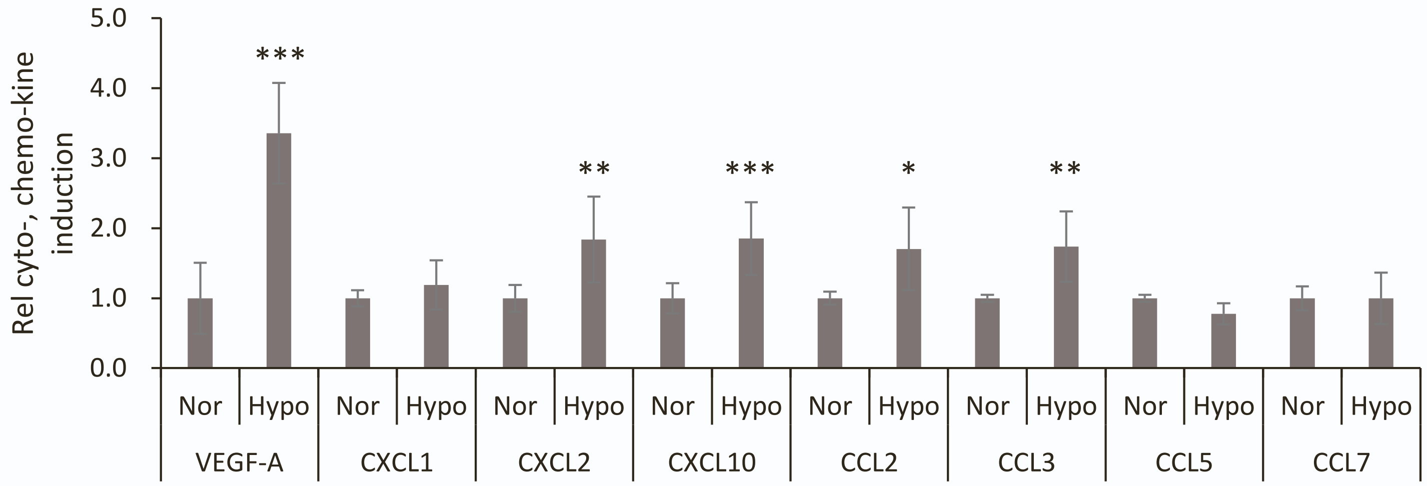

Figure S4. Relative secretion profiling of cytokines and chemokines from rMC1 cells under hypoxic conditions. Notably, the levels of CCL2, CCL3, CXCL2, CXCL10, and VEGF-A significantly increased under hypoxia. Results are presented as mean ± SD, with significant differences indicated (* $p < 0.05$, ** $p < 0.01$ and *** $p < 0.001$) based on three independent experiments. n≥7

Figure S5

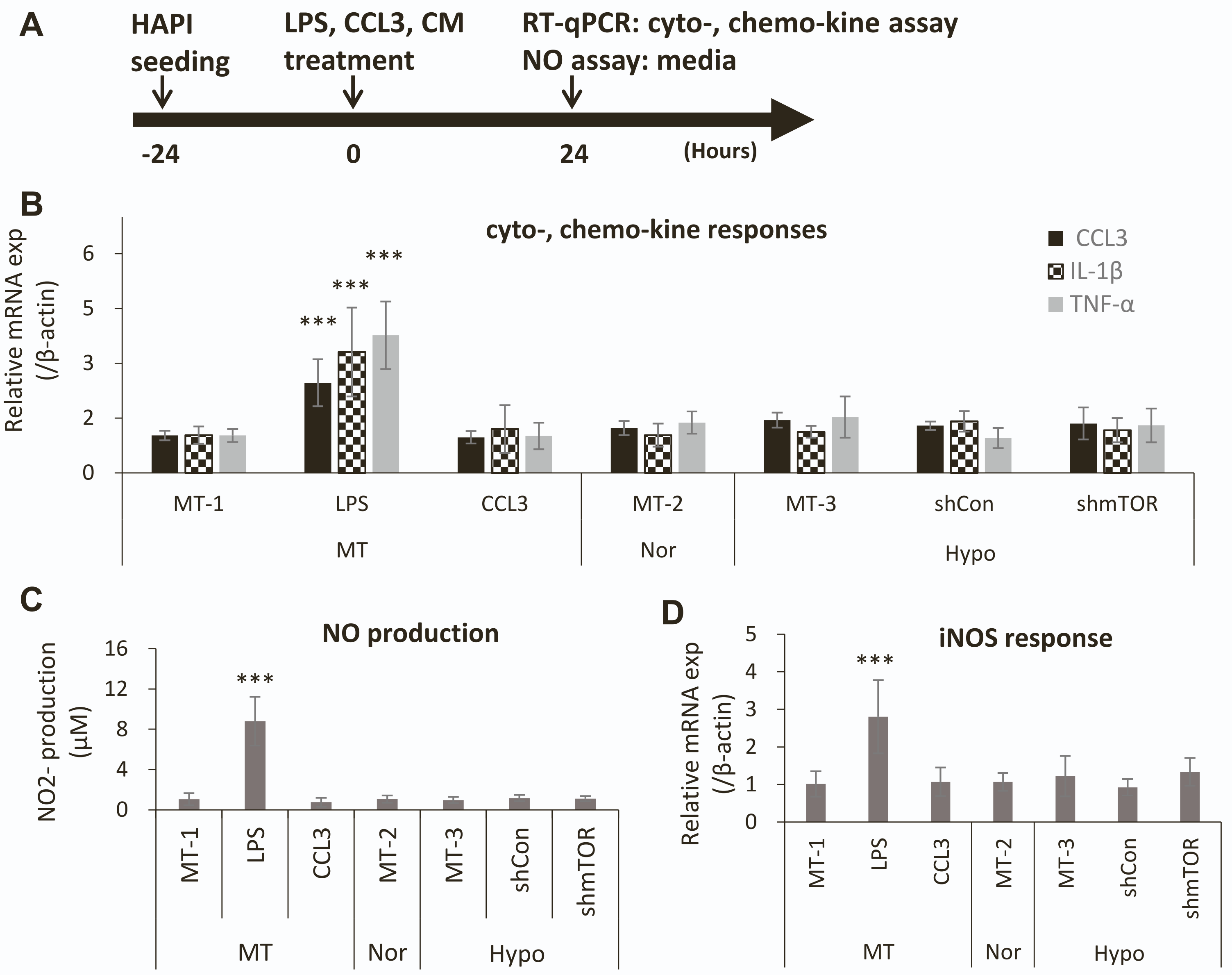

Figure S5. Responses of rat microglia cells following the exposure to CM from AAV2-shmTOR treated rMC1 cells under hypoxia. HAPI microglia cells were treated with LPS, CCL3, or CM. (A) Diagram outlining the experimental setup. (B) mRNA levels for CCL3, IL1-β, and TNF-α. (C) NO production. (D) iNOS mRNA level. Following the incubation with CM, there was no increase in the expression of various cytokines' mRNA or NO production. The results are presented as mean ± SD, with significant findings (*** $p < 0.001$) based on data from three experiments.

Supplemental Materials and Methods

Real-time quantitative polymerase chain reaction (RT-qPCR)

The total RNA preparation and RT-qPCR were conducted in accordance with the methodology described in the M & M. The primers used for CCL3, IL-1β, TNF-α and β-actin were as follows: CCL3, forward, 5'- AAGAGACCTGGGTCCAAGAA -3', reverse, 5'- GATTTGCAGGTGGCAGGAAT -3'; IL-1β, forward, 5'- GTTTGAGTCTGCACAGTTCC -3', reverse, 5'- AAGACACGGGTTCCATGGTG -3'; TNF-α, forward, 5'- TCGTAGCAAACCACCAAGC -3', reverse, 5'- TGCTGGTACCACCAGTTGG -3'; and β-actin, forward, 5'- TGAAGATCAAGATCATTGCTC -3', reverse, 5'- TGCTGGTACCACCAGTTGG -3'.

Tissue preparation and immunohistochemistry

The tissue preparation and immunohistochemistry were conducted according to the procedures described in the M & M.

Nitric oxide (NO) production

NO production was measured using the colorimetric Griess assay (Promega, Madison, WI) to assess nitrite concentration, a stable NO metabolite, in HAPI microglia cells. This was carried out after treatment with 1.0 μg/ml of LPS (Sigma-Aldrich), 100 ng/ml of CCL3 (Thermo Fisher Scientific), or conditioned media (CM) collected from rMC1 cells under various conditions. Absorbance readings were taken at 540 nm with a microplate reader.
