# Supplementary material for: mTOR downregulation promotes anti-inflammatory responses via the CCL3-CCR5 axis in hypoxic retinopathy
Source: Mol Ther Methods Clin Dev. 2024 Dec 31;33(1):101404. doi: 10.1016/j.omtm.2024.101404 (PMC11787640; doi:10.1016/j.omtm.2024.101404)
Supplement: Document S2. Article plus supplemental information [file mmc2.pdf]

Molecular Therapy
Methods & Clinical Development
Original Article

mTOR downregulation promotes anti-inflammatory responses via the CCL3-CCR5 axis in hypoxic retinopathy

Tae Kwon Moon,[1,2,8] Im Kyeung Kang,[1,2,8] Kyoung Jin Lee,[2,3] Ji Hyun Kim,[2,3] Hee Jong Kim,[4] A. Reum Han,[1,5] Ha-Na Woo,[3,5] Joo Yong Lee,[3,6] Jun-Sub Choi,[4] Keerang Park,[4] and Heuiran Lee[3,7]

[1]Asan Medical Institute of Convergence Science and Technology, Asan Medical Center, University of Ulsan College of Medicine, Seoul, Republic of Korea; [2]Department of Microbiology, University of Ulsan, College of Medicine, Seoul, Republic of Korea; [3]Bio-Medical Institute of Technology, University of Ulsan College of Medicine, Seoul, Republic of Korea; [4]Institute of New Drug Development Research, CdmoGen Co., Ltd., Seoul 05854, Republic of Korea; [5]Department of Biochemistry & Molecular Biology, University of Ulsan College of Medicine, Seoul, Republic of Korea; [6]Department of Ophthalmology, Asan Medical Center, College of Medicine, University of Ulsan, Seoul, Republic of Korea; [7]Department of Microbiology, Asan Medical Center, College of Medicine, University of Ulsan, Seoul, Republic of Korea

Hypoxic retinopathies, including diabetic retinopathy, are major contributors to vision impairment, mainly due to accelerated angiogenesis and inflammation. Previously, we demonstrated that AAV2-shmTOR, effective across distinct species, holds therapeutic promise by modulating the activated mTOR pathway, yet its mechanisms for reducing inflammation remain largely unexplored. To investigate AAV2-shmTOR's impact on atypical inflammation in these conditions, we employed an *in vivo* model of oxygen-induced retinopathy and an *in vitro* model using rMC1 Müller cells. AAV2-shmTOR notably decreased mTOR expression in rMC1 cells under hypoxic conditions, as verified by co-staining for mTOR and glial fibrillary acidic protein (GFAP). It effectively interrupted the activation of mTOR signaling triggered by hypoxia. It diminished the secretion of CCL3 from rMC1 cells, consequently reducing microglial migration in response to conditioned media from AAV2-shmTOR-treated rMC1 cells. Notably, the virus lowered CCL3 expression in Müller cells and reduced the presence of CCR5-positive microglia *in vivo*, indicating its effectiveness in targeted inflammation management via the CCL3-CCR5 pathway. These findings thus highlight the potential of AAV2-shmTOR to exert anti-inflammatory effects by influencing the mTOR and subsequent CCL3-CCR5 pathways in hypoxic retinopathies, presenting a novel therapeutic approach for retinal diseases marked by hypoxia-driven inflammation.

INTRODUCTION

Retinopathy, a disease affecting the retina, can cause irreversible vision loss and thus underscores the need for effective treatments. The most common forms, diabetic retinopathy (DR) and age-related macular degeneration (AMD), both involve retinal neovascularization (RNV).[1,2] RNV can lead to complications like vitreous hemorrhage, inflammation, tissue edema, and fibrotic scar formation, causing irreversible retinal damage and blindness.[3] Vascular endothelial growth factor (VEGF) plays a crucial role in RNV, and treatments like ranibizumab (Lucentis), aflibercept (Eylea), and bevacizumab (Avastin) are commonly used anti-VEGF agents.[4] Administered through direct intraocular injection, they have caused a breakthrough in preserving and sometimes improving vision.[5] However, the anti-VEGF treatments necessitate repeated injection, vary in patient response, and can cause complications such as infections and retinal detachment.[6,7] Their limited action duration adds economic and medical burdens, driving the need for more durable and practical solutions.[8]

In a rapidly evolving field, adeno-associated virus 2 (AAV2)-based gene therapy may offer a promising solution for retinopathies. Addressing these diseases' underlying genetic causes or contributing factors could provide long-lasting effects.[9,10] Current efforts are focused on delivering genes that can modulate therapeutic proteins directly within the eye, potentially eliminating the need for repeated treatments.[11,12] These strategies include introducing genes that create anti-VEGF compounds internally or focusing on the complement factor pathway to prevent abnormal blood vessel formation or reduce inflammation.[13,14]

We previously demonstrated the novel and promising potential of a therapeutic AAV2 expressing small interfering mTOR with universal cross-species specificity, including human, murine species, and monkey (AAV2-shmTOR),[15] across various types of retinopathies using animal models such as choroidal neovascularization (CNV),

Received 27 June 2024; accepted 28 December 2024;
https://doi.org/10.1016/j.omtm.2024.101404.

[8]These authors contributed equally

Correspondence: Keerang Park, Institute of New Drug Development Research, CdmoGen Co., Ltd., Seoul 05854, Republic of Korea.
E-mail: keerang.park@gmail.com
Correspondence: Heuiran Lee, Bio-Medical Institute of Technology, University of Ulsan College of Medicine, Seoul, Republic of Korea.
E-mail: heuiran@amc.seoul.kr

Published by Elsevier Inc. on behalf of The American Society of Gene and Cell Therapy.
This is an open access article under the CC BY license (http://creativecommons.org/licenses/by/4.0/).

oxygen-induced retinopathy (OIR), and diabetes-related retinopathy (DR) murine models.[16–18] The therapeutic effects were confirmed by a notable reduction in abnormal angiogenesis and leaky vessel development.[17,18] Virus administration interfered with inflammatory cell penetration into retinal tissue, as lower CD11b and F4/80 immunohistochemistry (IHC) staining showed.[17,18] The virus possessed anti-apoptotic properties and resultant neural cell maintenance in the ganglion layer, and the integrity of the retinal layer was comparatively preserved.[13,16] Furthermore, the latest study has revealed that the mitigating impact of AAV2-shmTOR on pathological RNV is derived from its ability to inhibit the VEGF-stimulated state of endothelial cells.[19] The virus efficiently inhibited the angiogenic potential of endothelial cells by maintaining a normal level of cell proliferation and enhancing cellular integrity by maintaining tight junctions between the cells. However, the detailed mechanism by which AAV2-shmTOR alleviates inflammation has yet to be determined.

Hypoxia, characterized by an inadequate oxygen supply to tissues, is a common pathological feature in various retinopathies.[20,21] It triggers the production of proangiogenic factors like VEGF and initiates inflammatory responses, including the release of cytokines. Müller cells become activated in these conditions, essential for maintaining the retina's structural and functional integrity. This activation can either protect against or accelerate the progression of retinopathy.[22,23] In addition, Müller cells and microglia are both integral to the inflammatory response[24] in the retina. Müller cells can release cytokines and chemokines that activate microglia, while activated microglia can induce Müller cell gliosis, exacerbating inflammation and potentially contributing to retinal pathology.[25] In this study, we explored the effects of AAV2-shmTOR on Müller cells in hypoxic conditions, employing both *in vitro* and *in vivo* approaches to assess its role in mitigating pathological inflammation in retinopathy. The results showed a significant decrease in inflammatory markers and reduced mTOR activation in Müller cells under hypoxia. The virus lowered CCL3 and CCR5 expression in hypoxic retinas, pointing to a potential anti-inflammatory mechanism.

RESULTS

mTOR upregulation in activated Müller cells was reduced by AAV2-shmTOR administration in an OIR model

The affinity of AAVs for Müller cells in hypoxic retinas was evaluated using the OIR model.[26] This model involves exposing newborn pups to alternating oxygen concentrations, which results in altered neovascularization and leads to retinal hypoxia (Figure S1A). The administration of intravitreal AAV2-GFP in a rat OIR model revealed that Müller cells have a high affinity for AAV2, as indicated by the co-expression of GFP and glutamine synthetase (GS) (Figures S1B and S1C). Under these OIR conditions, a significant increase in mTOR expression was observed, particularly within the expanded population of glial fibrillary acidic protein (GFAP)-positive Müller cells that exhibited tropism toward AAV2 (Figures 1, S2, and S3). However, unlike AAV2-shCON, the administration of AAV2-shmTOR, which expresses small interfering mTOR with cross-species specificity (Figure 1B), effectively reduced this increase in mTOR expression. The result thus indicates that the intravitreal delivery of therapeutic AAV2 encoding shmTOR can successfully suppress the upregulation of mTOR in activated Müller cells within the hypoxic environment of the OIR model.

The mTOR signaling pathway was upregulated in rMC1 cells under hypoxic conditions and was attenuated following AAV2-shmTOR treatment

The extent of mTOR knockdown in rat Müller cells (rMC1) was confirmed, as expected, to be dose dependent on AAV2-shmTOR treatment, both at the mRNA and protein levels (Figures 2A–2D). rMC1 cells were exposed to hypoxia in a chamber with 2% oxygen during cell culture (Figure 2E). Western blot analysis assessed the impact of hypoxia and AAV2-shmTOR treatment on the mTOR signaling pathway. Hypoxia increased mTOR signaling, shown by higher levels of mTOR and phosphorylated p70S6K and AKT in mTOR complex 1 (mTORC1) and mTORC2, respectively (Figure 2F). The group treated with AAV2-shmTOR showed a significant decrease in mTOR levels compared to the hypoxia control and AAV2-shCON groups (Figure 2G) and reduced phosphorylation ratios of p70S6K and AKT (Figures 2H and 2I). These findings show that AAV2-shmTOR treatment effectively suppressed mTOR expression and downstream signaling in mTORC1 and mTORC2.

CCL3 secretion increased under hypoxic conditions and was reduced in rMC1 cells by AAV2-shmTOR treatment

To investigate how AAV2-shmTOR affects the secretion patterns of various cytokines and chemokines by Müller cells under hypoxic conditions, we used a cytokine/chemokine multiplex assay to analyze 23 representative factors. Among these factors, CCL2, CCL3, CXCL2, CXCL10, and VEGF-A significantly increased under hypoxic conditions (Figure S4). Moreover, both CCL3 and VEGF-A mRNA expression and secretion decreased significantly after shmTOR virus treatment. Notably, while VEGF levels remained above normoxic levels, CCL3 levels nearly normalized (Figure 3).

AAV2-shmTOR-treated rMCl cells effectively reduced the CCL3-dependent migration of microglia cells

While VEGF-A has a role in inflammation, its primary functions in retinopathy are widely recognized as angiogenesis and increased vascular permeability. In this context, our previous study demonstrated that inhibiting VEGF-A expression using AAV-shmTOR plays a crucial role in reducing pathological angiogenesis.[19] Building on this, we shifted our focus to the chemokine CCL3, also known as macrophage inflammatory protein-1α (MIP-1α), which is known for recruiting and activating immune cells, such as macrophages, thereby contributing to chronic inflammation in retinal tissue.[27,28] We assessed the effect of AAV2-shmTOR on the migratory capabilities of HAPI microglia cells using conditioned media (CMs) from rMC1 cells treated under hypoxic conditions with mock treatment, AAV2-shCON, or AAV2-shmTOR (Figure 4A). We observed that microglia cell transmigration was enhanced with media from hypoxic

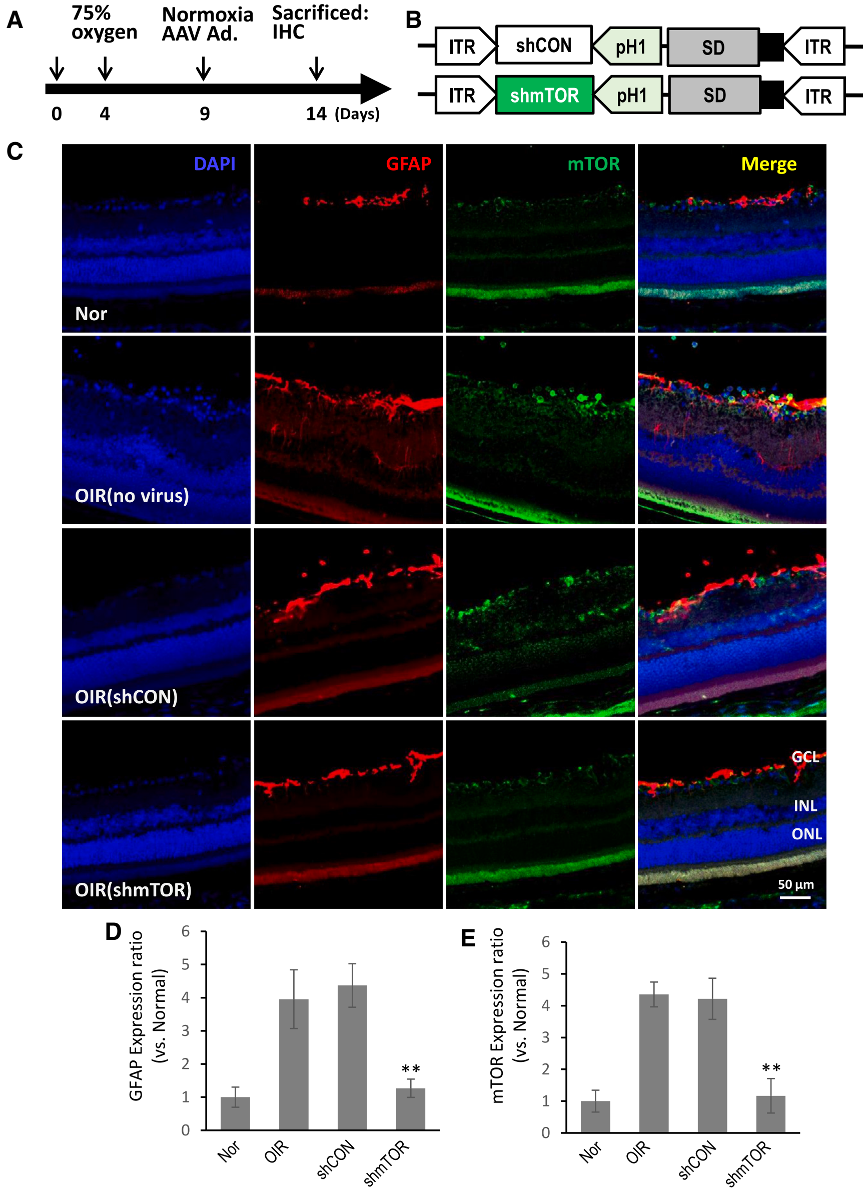

Figure 1. mTOR modulation in Müller cells by AAV2-shmTOR

(A) Diagram outlining the experimental setup. (B) Schematic overview of AAV2-shCON and AAV2-shmTOR. (C) Immunohistochemistry targeting GFAP and mTOR. (D) Quantified analysis of Müller cell activation. (E) Quantified analysis of mTOR expression. These findings reveal that mTOR is upregulated in Müller cells under OIR conditions and downregulated following AAV2-shmTOR treatment. The results of the quantified analysis results are presented as mean ± SD, with significant findings (**$p < 0.01$) based on data from three experiments. $n \geq 3$ (three eyes from three mice). Nor, normal; GCL, glial cell layer; INL, inner cell layer; ONL, outer cell layer; OIR, oxygen-induced retinopathy.

conditions but was notably reduced with AAV2-shmTOR treatment, unlike the shCON virus, which did not inhibit migration (Figures 4B and 4C). Additionally, under these experimental conditions, we detected no significant alterations in inflammatory cytokine levels or nitric oxide (NO) production in the microglia cells (Figure S5). These findings suggest that shmTOR specifically decreases CCL3 expression under hypoxic conditions, thus regulating CCL3-dependent microglia migration.

CCL3 and CCR5 expression is upregulated in the hypoxic retina and reduced by AAV2-shmTOR treatment

As shown in Figure 1, we observed Müller cell activation via GFAP staining in the retina after hypoxia. Here, we performed this experiment to observe CCL3 expression in Müller cells of hypoxic retina. The expression of CCL3 and its corresponding receptor, CCR5, on the plasma membrane was immunochemically monitored in the hypoxic retina. CCL3 and Müller cell-specific GS staining showed an apparent increase in CCL3 expression under hypoxic conditions, which was followed by a subsequent decrease after AAV2-shmTOR administration (Figure 5). Similarly, staining for CCR5 and the microglia cell-specific Iba1 revealed a marked increase in CCR5 expression under hypoxic conditions, which then decreased in microglia cells following shmTOR virus treatment (Figure 6). These findings imply the therapeutic potential of AAV2-shmTOR through the CCL3-CCR5 axis in hypoxic conditions.

DISCUSSION

This study provides evidence that in hypoxic retinal disorders, AAV2-shmTOR-mediated inhibition of the mTOR pathway reduces activated microglial cell activity via Müller cells, leading to diminished inflammatory pathologies. The AAV2-shmTOR virus has been observed to reduce inflammatory markers, including CCL3 and VEGF-A, by effectively via the mTOR and CCL3-CCR5 pathways. It inhibits microglial cell migration, thereby decreasing inflammation and potentially preventing the progression of retinal diseases (Figure 7). The findings highlight that AAV2-shmTOR not only targets the mTOR pathway but also significantly reduces the secretion of CCL3 and the presence of CCR5-positive microglia. We recently discovered that AAV2-mTOR effectively inhibits the proliferation and migration of endothelial cells in response to elevated VEGF levels in hypoxic retinas, thereby mitigating aberrant angiogenic effects.[19] This study also suggests a direct role of the shmTOR virus in controlling inflammation via CCL3-CCR5-specific pathways. The present findings thus reveal the fundamental process by which AAV-shmTOR significantly reduces pathological inflammation in

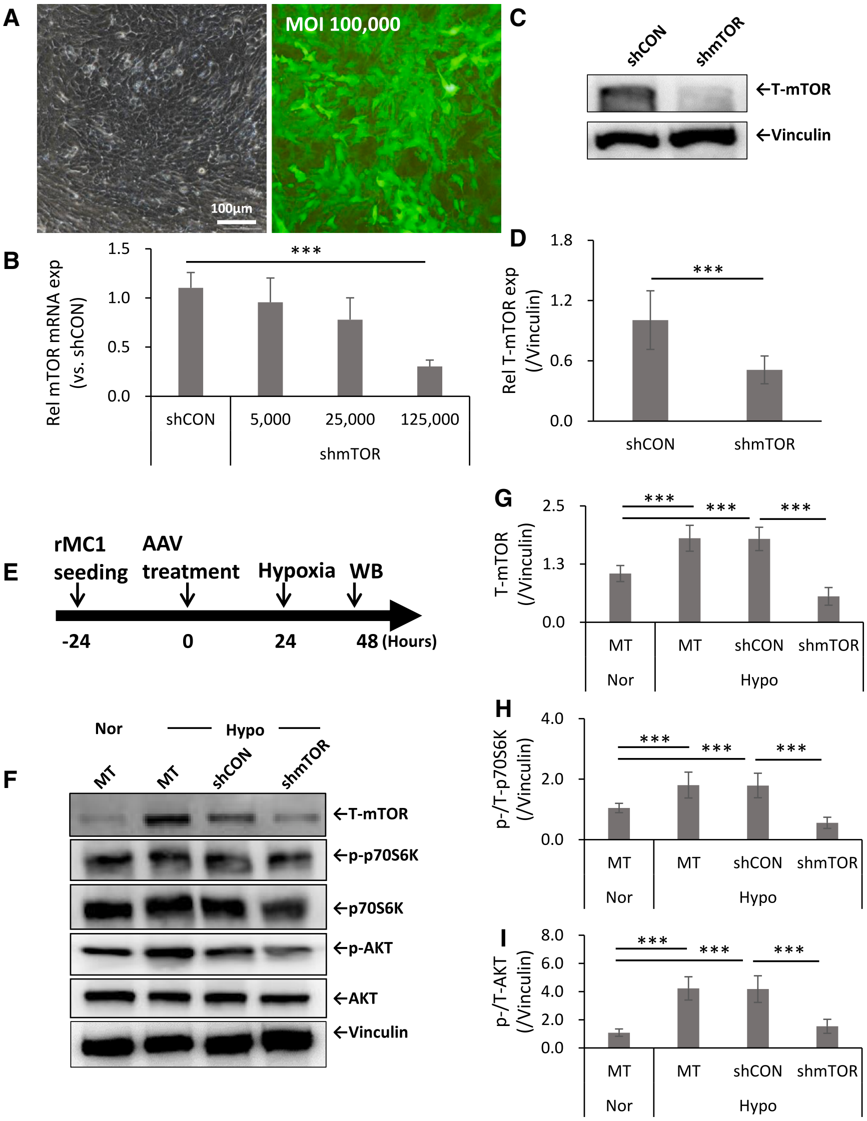

Figure 2. mTOR knockdown and mTOR signaling regulation by AAV2-shmTOR treatment in rMC1 cells under hypoxic conditions

The expression levels of mTOR and its downstream targets were assessed using real-time qPCR and western blot analysis. (A) Transduction efficiency by GFP expression following AAV2-GFP treatment for 48 h. (B) Dose-dependent mTOR mRNA knockdown effects of AAV2-shmTOR treatment as analyzed by real-time qPCR. (C and D) mTOR protein knockdown effects of AAV2-shmTOR treatment as analyzed by a western blot. (E) Experimental schematic of western blot. (F) Expression patterns for proteins associated with mTOR signaling, such as mTOR, p70S6K, and AKT. (G–I) Quantified data for these proteins. Vinculin was used as a loading control. The results indicate a decrease in the phosphorylation of mTOR downstream factors in the AAV2-shmTOR group under hypoxic conditions, with data shown as mean ± SD and statistically significant differences marked (***$p < 0.001$) from at least three experiments. MT, mock treated (no virus); Nor, normoxic normal condition; Hypo, hypoxia.

CNV, OIR, and DR animal models, representing diverse types of retinopathies.[16–18]

Dysregulation of the mTOR signaling pathway is frequently observed in several forms of retinopathy, including DR and AMD.[29,30] mTOR function is mediated by two distinct multimeric complexes in which mTOR is the common major player: mTORC1 and mTORC2.[31] Activation of mTOR plays a crucial role in promoting harmful processes like neovascularization, inflammation, and neuronal damage, which can impair vision. Due to their capability to infect non-dividing cells, their persistent therapeutic gene expression, and their excellent safety profile, AAV vectors are particularly effective for targeting retinal cells.[32,33] Luxturna, the first FDA-approved gene therapy for an inherited disease, utilizes AAV2 vectors to deliver a normal copy of the RPE65 gene to retinal cells, addressing mutations that cause Leber congenital amaurosis (LCA).[34,35] We have previously engineered an AAV2 vector encoding shmTOR,[15] which precisely targets and silences the mTOR gene, thereby diminishing the heightened mTOR signaling seen in various retinopathy models in animals.[16–18] This AAV2-shmTOR effectively produces simTOR in the targeted cells and demonstrates efficacy across multiple species, an essential step for preclinical and clinical research advancements. It contributes to the progress in developing small interfering RNA (siRNA)-based therapeutics. Tang et al. have utilized siRNA to target Janus kinase 1 (JAK1) in various species, illustrating that the dysregulation of JAK1 plays a role in numerous immune disorders.[36] When administered via intravitreal injection, the therapeutic effects of AAV2-shmTOR have been proven to mitigate adverse processes such as neovascularization, inflammation, and neuronal damage.

AAV2-based gene therapy is a promising approach for treating various retinal diseases, such as LCA, choroideremia, and AMD.[9,10] While rapamycin, a potent mTOR inhibitor, could potentially be applied, but AAV2-shmTOR offers several advantages. Unlike rapamycin, which requires systemic administration and affects the entire body, AAV2-shmTOR can be delivered directly to the eye, minimizing side effects and providing sustained mTOR silencing in ocular tissues with a single injection.[16,19] The biodistribution study in non-human primates (NHPs) showed that AAV2-shmTOR-SD is primarily localized in the vitreous humor and around the injection site of the treated eye, with virus levels in non-ocular tissues below quantifiable limits (Table S1). Thus, AAV2-shmTOR offers more specific, localized, and durable treatment, making it a promising option for

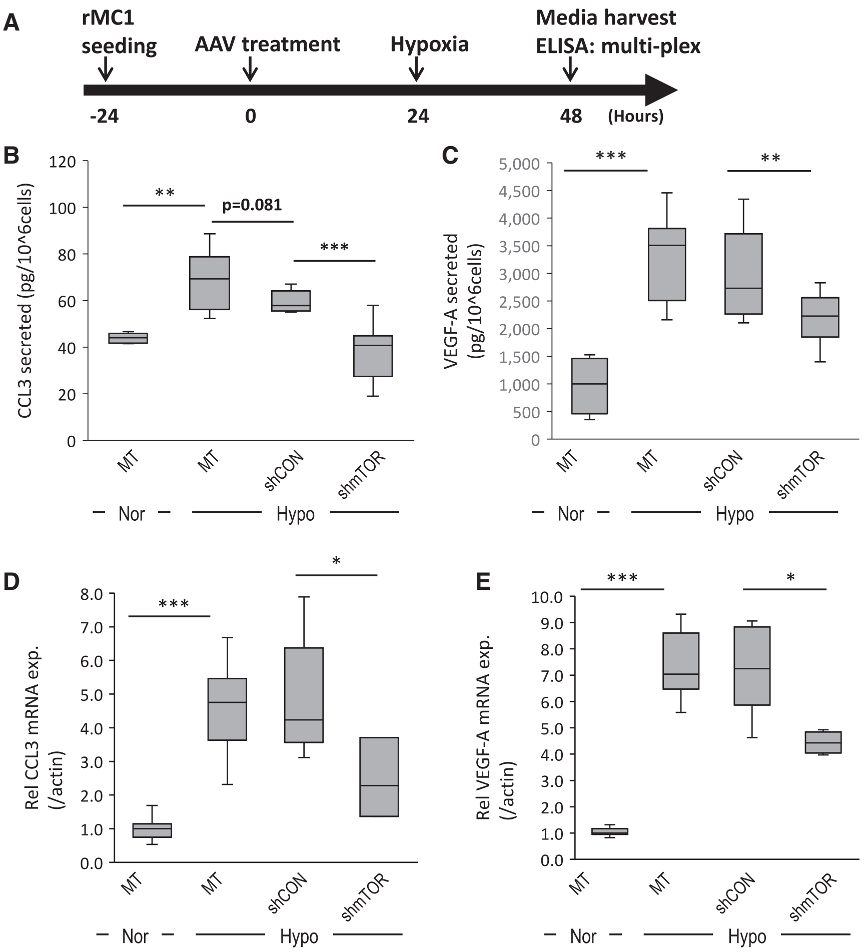

Figure 3. Chemokine profiling expressed from rMC1 following AAV2-shmTOR treatment under hypoxic conditions

(A) Experimental schematic for multiplex ELISA. (B) CCL3 protein secretion patterns. (C) VEGF-A protein secretion patterns. (D) CCL3 mRNA expression patterns. (E) VEGF-A mRNA expression patterns. Under hypoxic conditions, both the secretion and mRNA expression of CCL3 and VEGF-A were elevated compared to normoxic conditions, while AAV2-shmTOR treatment reduced these levels of both CCL3 and VEGF-A. The results are presented as mean ± SD, with significant differences indicated (**$p < 0.01$ and ***$p < 0.001$) from three experiments. MT, mock treated (no virus); Nor, normoxic normal condition; Hypo, hypoxia.

long-term management of retinopathy. Compared to other viral vectors, such as adenovirus, lentivirus, and retrovirus, non-pathogenic AAV has several advantages in terms of safety and efficacy. However, there are still some safety concerns associated with AAV vectors, such as immunogenicity and toxicity. Nevertheless, our recent results from a 13-week toxicity study and immune response evaluation of AAV2-shmTOR in NHPs indicated no observed toxicity in major organs, including the eyes, brain, and liver (Table S1). Furthermore, while there was an increase in T cell immune response following intravitreal injection, this response diminished rapidly, with no significant immune response detected subsequently, indicating the promising potential of AAV2-shmTOR as a novel treatment. Lastly, despite intravitreal injection of AAV2-shmTOR, there still remains a possibility that the virus could migrate to other organs. Therefore, conducting a biodistribution analysis is crucial to identify any potential off-target effects or side effects. Through the recent completion of this analysis, we were able to elucidate the compelling therapeutic potential of AAV2-shmTOR (Table S1).

Müller cells, the primary glial cells in the highly differentiated retina, are crucial for maintaining structural and functional integrity. They constantly interact with neurons, endothelial cells, and microglia to preserve the retina's architecture and function.[37,38] Under hypoxic conditions, Müller cells become reactive, changing their morphology and function, which can lead to pathological inflammation via the secretion of cytokines and chemokines.[39] In addition to secreting VEGF, which promotes RNV, Müller cells release chemokines such as CCL2, CCL3, CXCL2, and CXCL10 (data not shown). In another study, besides the cytokines identified above, interleukin (IL)-17A or IL-33 was also upregulated in Müller cells, particularly in DR.[40,41] The chemokine CCL3, also known as MIP-1α, recruits immune cells, including T cells, monocytes, and microglia. CCR5, a receptor expressed on microglia, has been known to bind with CCL3.[42,43] mTOR can modulate inflammation by influencing cytokine production and other mediators. Depending on the context and specific immune cell types, its activation either promotes or suppresses inflammation.[30,44,45] In macrophages, mTORC1 activation has shown the production of pro-inflammatory cytokines. Notably, AAV2-shmTOR negatively regulated the release of CCL3 in Müller cells (Figures 3 and 5), reduced the migration of microglia cells (Figure 4), and decreased the presence of CCR5-positive microglia in hypoxic retina (Figure 6). As far as we are aware, this is the first report demonstrating the involvement of the CCL3-CCR5 axis in targeted inflammation regulation, modulated by the mTOR pathway, within the interactions between Müller cells and microglia in hypoxic retina.

While this study offers promising insights into the underlying mechanisms of the anti-inflammatory effects of AAV2-shmTOR, it also highlights the need for further research. One limitation is that the research was conducted solely on OIR models representing certain types of hypoxic retinopathy. Although hypoxic conditions are a common feature and crucial in the progression of both AMD and DR, the detailed mechanisms by which they contribute to each disease

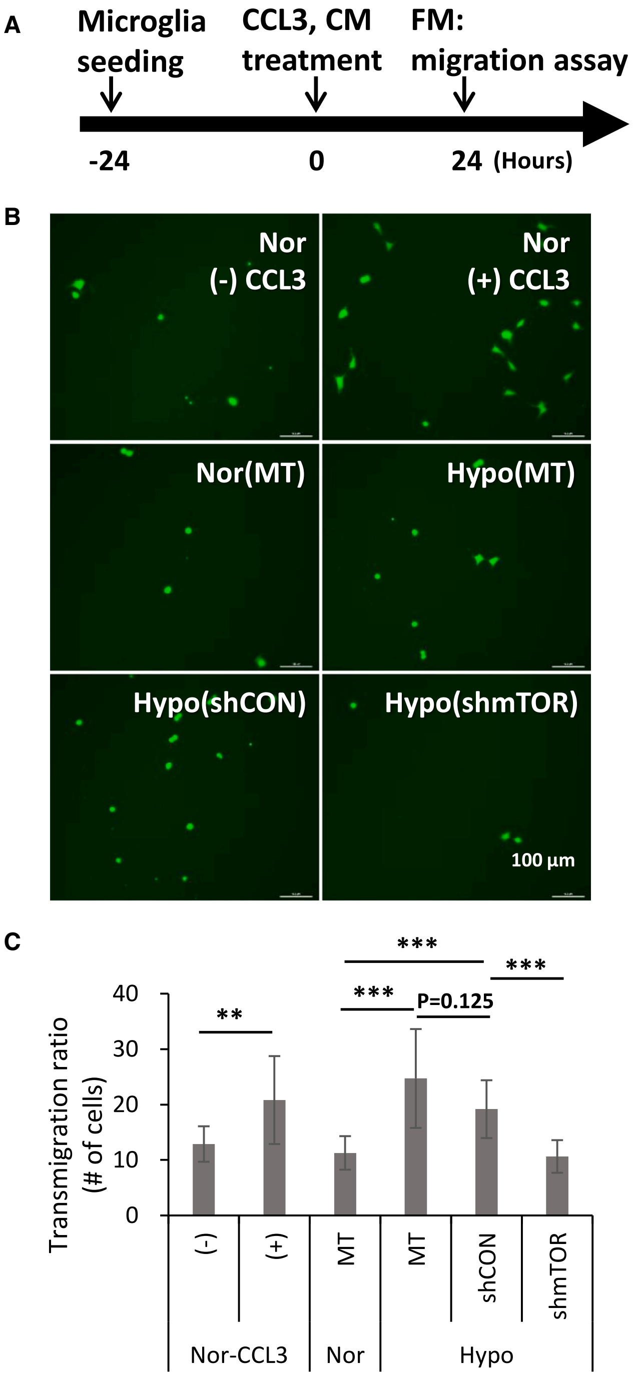

Figure 4. Transmigration of microglia cells after exposure to conditioned media from AAV2-shmTOR-treated rMC1 under hypoxia

Microglial cells expressing GFP in a transwell insert were treated with conditioned media collected from rMC1 cells. The degree of microglial transmigration across the insert was evaluated by counting GFP-positive cells using fluorescence microscopy. (A) Experimental schematic of migration assay. (B) Transmigration patterns of microglia cells under various conditions. (C) Quantification of transmigration degree for each condition. Quantification data are presented as mean ± SD, with significance noted (*$p < 0.05$ and **$p < 0.01$) from three independent experiments. The CCL3-treated group was used as a positive control. CM, conditioned media; FM, fluorescence microscopy; MT, mock treated; Nor-CCL3, normoxic normal condition; (–), not treated with CCL3; (+), treated with CCL3; Hypo, hypoxia.

vary. In DR, chronic hyperglycemia damages the blood vessels of the retina, causing them to leak or become blocked, leading to ischemia and subsequent hypoxia.[46,47] In advanced stages of AMD, particularly in neovascular AMD, the choroid (a layer of blood vessels behind the retina) tries to grow new blood vessels into the retina to circumvent blockages or areas of poor perfusion, driven by hypoxia-induced factors like VEGF.[48,49] In this context, the shmTOR virus in modulating the CCL3-CCR5 axis in CNV or DR animal models needs further exploration, which is currently underway.

In conclusion, AAV2-shmTOR represents a promising advance in gene therapy for retinal diseases, offering a targeted approach to mitigate inflammation and neovascularization, pivotal elements in the progression of retinopathy. The study establishes a basis for the therapeutic use of gene therapy in managing retinal hypoxia and related pathologies, offering an alternative to traditional treatments that require frequent intravitreal injections, such as anti-VEGF therapies. The proven effectiveness of AAV2-shmTOR in various animal models of retinopathy also points to its potential for broad application in clinical settings. However, the findings also indicate a need for further exploration into diverse models of hypoxic retinopathy to understand this treatment's versatility and limitations.

MATERIALS AND METHODS

Cell culture

rMC1 (Kerafast, Boston, MA), rat microglia (ABM, New York, NY), and HAPI (Sigma-Aldrich, St. Louis, MO) cells were cultured in Dulbecco's modified Eagle medium (Thermo Fisher Scientific, Waltham, MA) supplemented with 10% fetal bovine serum (Corning, Corning, NY), 2 mM GlutaMAX-1 (Thermo Fisher Scientific), and penicillin (100 IU/mL)/streptomycin (50 µg/mL) (Corning). The cells were incubated at 37°C under a humidified 5% carbon dioxide atmosphere.

Preparation of AAV2 vectors

The AAV2-shmTOR-SD vector, as previously detailed,[50] was generated by removing the GFP expression cassette, including the CMV promoter, GFP reporter gene, and polyadenylation signal, from the viral plasmid p AAV2-shmTOR-SD and inserting a fragment of the human UBE3A gene. Similarly, the control virus AAV-shCON-SD was prepared. The sequence information for shmTOR and shCON, driven by the pH1 polymerase III promoter (Figure 1B), was previously documented in the original paper where AAV2-shmTOR was initially developed.[15] All the AAV2 pseudotype vectors were supplied by CdmoGen (Cheongju, Republic of Korea). Viral titers were determined using real-time qPCR with the AAVpro Titration Kit from Takara (San Jose, CA). The rMC1 cells were infected with AAV2-shCON-SD or AAV2-shmTOR-SD at 1.0×10^5 MOI for 48 h.

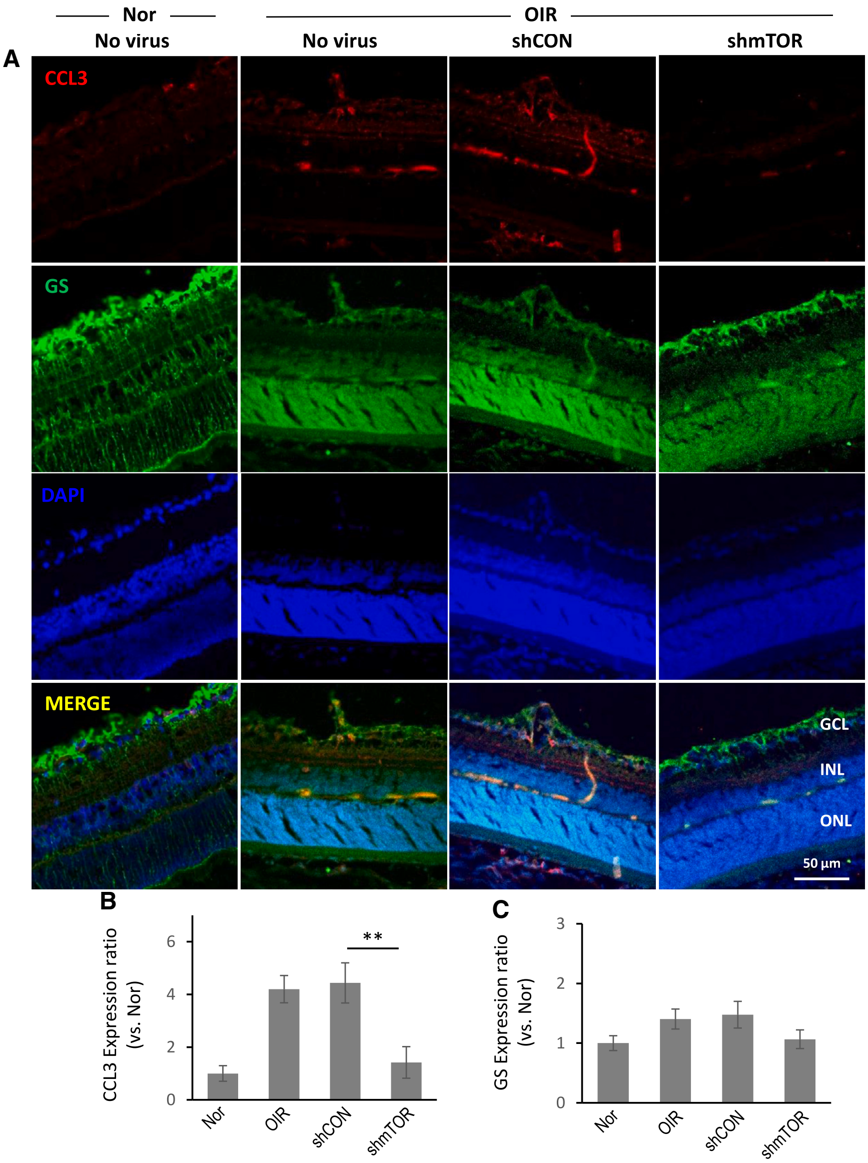

Figure 5. CCL3 expression in an OIR model

CCL3 expression in Müller cells in retinal tissues was examined by immunohistochemically using primary antibodies against CCL3 and GS. (A) Immunohistochemistry specific to CCL3 and GS. (B) Quantitative analysis of CCL3 expression (C) Quantitative analysis of GS expression. CCL3 expression was upregulated under OIR conditions and attenuated by AAV-shmTOR administration in GS-positive Müller cells. Data are presented as mean ± SD, with significance (**$p < 0.01$) from three experiments. GS, glutamine synthetase.

Animal care and rat model of OIR

Sprague-Dawley rats from The Orient Bio (Sungnam, Korea) were utilized in this study, which received approval from the Asan Institute for Life Sciences' Internal Review Board for Animal Experiments (University of Ulsan, College of Medicine). The study complied with the guidelines for animal care and experimental procedures by the Association for Research in Vision and Ophthalmology. The well-established protocol for mice set forth by Smith et al. was generally followed to generate the rat OIR model. Following the Smith et al. protocol for mice,[17,51,52] we adapted it for our rat OIR model. Due to lower neovascularization levels in rats than mice, we exposed Sprague-Dawley rat pups to hyperoxia on post-natal day (P)4 to leverage their underdeveloped retinal vasculature for enhanced vaso-obliteration. Neovascularization intensity depends on the degree of vaso-obliteration. On P9, we returned the pups to normoxia and administered treatments—intravitreal injections of their respective virus vectors, mock treatments, or no treatment—before euthanizing them on P14. Before the intravitreal injections, rats were anesthetized with an intraperitoneally administered 4:1 mixture of Zoletil (40 mg/kg) from Virbac (Carros Cedex, France) and Rompun (5 mg/kg) from Bayer Healthcare (Leverkusen, Germany). Their pupils were dilated using Mydrin-P (0.5% tropicamide and 2.5% phenylephrine) from Santen (Osaka, Japan). Subsequently, they received intravitreal injections of 1 μL of virus vectors at a 5.0×10^{10} viral genome/mL concentration. Three eyes from at least three mice were used in each experiment.

Real-time qPCR

TRIzol reagent (Invitrogen) was used to prepare total RNA from the cells. After that, Superscript III (Invitrogen) was used to reverse transcribe cDNA from RNA, and a SYBR Green kit (Invitrogen) was used to analyze mRNA levels. The following primers were used for PCR amplification purposes. mTOR (forward, 5′-TGAAGGCCACTCTCTGACCC-3′; reverse, 5′-CCATGCGGATCTCCTTGTGC-3′); CCL3 (forward, 5′-AAGAGACCTGGGTCCAAGAA-3′; reverse, 5′-GATTTGCAGGTGGCAGGAAT-3′); VEGF-A (forward, 5′-CTGCTGTACCTCCACCATGC-3′; reverse, 5′-CAATAGCTGCGCTGGTAGAC-3′); and β-actin (forward, 5′-TGAAGATCAAGATCATTGCTC-3′; reverse, 5′-TGCTTGCTGATCCACATCTG-3′).

Migration assay

Rat microglia cells expressing the GFP of 30,000 cells were added to the top well of each transwell insert (#35377, Falcon, NY, MA) with an uncoated filter with 8 μm diameter holes. The bottom wells contained only the medium. After 1 h, the cells were treated with

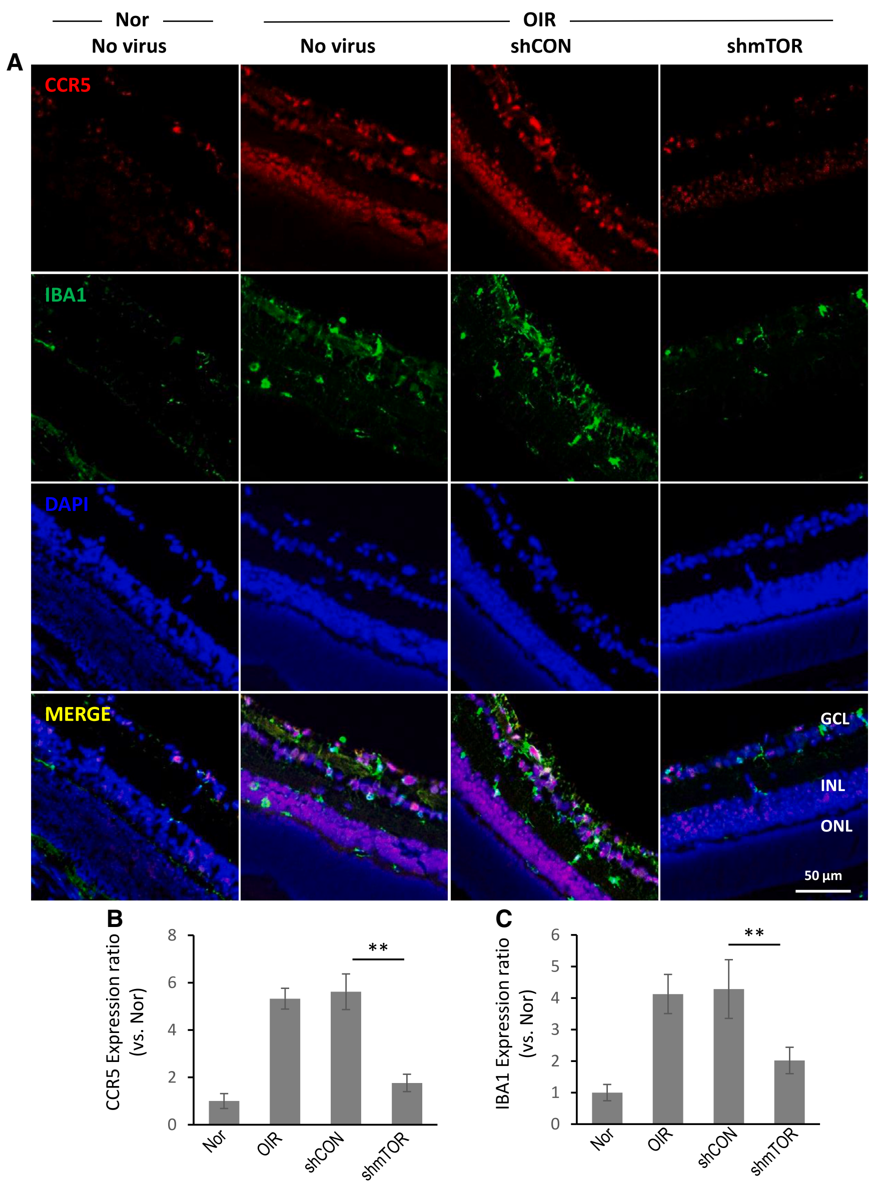

Figure 6. Expression pattern of CCR5 in an OIR model

CCR5 expression in microglia cells in retinal tissues was examined by immunohistochemically using primary antibodies against CCR5 and Iba1. (A) Immunohistochemistry specific to CCR5 and Iba1. (B) Quantitative analysis of CCR5 expression. (C) Quantitative analysis of Iba1 expression. CCR5 expression was upregulated in OIR condition and attenuated by AAV2-shmTOR administration in Iba1-positive microglia cells. Data are presented as mean ± SD, with significance (**$p < 0.01$) from three experiments.

100 ng/mL of CCL3 (Thermo Fisher Scientific) or CMs harvested from rMC1 cells at various conditions and incubated for 24 h. The number of cells expressing GFP that migrated downward was counted using the LSM 710 fluorescence confocal microscope (Carl Zeiss Microscopy, Jena, Germany). Images were captured using Zeiss Zen software (black edition, Carl Zeiss Microscopy). For each experiment, one well was used, and more than three independent studies were conducted. Five images were randomly captured from each well.

Western blot

Whole-cell lysates were prepared, and proteins were separated on sodium dodecyl sulfate (SDS)-polyacrylamide gels before being transferred to polyvinylidene fluoride (PVDF; Thermo Fisher Scientific) membranes. The membranes were blocked using Tris-buffered saline (TBS) with 0.1% Tween 20 and 5% (w/v) bovine serum albumin (Cytiva, MA) and then incubated with various primary antibodies (Abs) and suitable secondary Abs (#111-035-003 for rabbit, #315-035-003 for mouse, Jackson ImmunoResearch Lab, West Grove, PA). Bands were visualized using an enhanced chemiluminescence (ECL) system. Primary Abs for mTOR (1:1,000, #2983, Cell Signaling Tech., Danvers, MA), p70S6K 1:1,000, (AF8962, R&D Systems, Minneapolis, MN), phospho-p70S6K (1:1,000, AF8963, R&D Systems), AKT (1:1,000, #4691, Cell Signaling Tech.), and phospho-AKT (1:1.000, #4060S, Cell Signaling Tech.) were applied. Vinculin (1:1,000, MAB6896, R&D Systems) served as the normalization control. The secondary Abs were utilized in the experiment with a dilution factor of 1:1,000. The primary Ab was incubated overnight, and the secondary Ab was incubated for 1 h at 4°C.

Tissue preparation and IHC

After an intracardial perfusion with 0.1 M PBS containing 150 U/mL heparin and infusion with 4% paraformaldehyde (PFA; Thermo Fisher Scientific) in 0.1 M phosphate buffer (PB), the eyes were removed and fixed in 4% PFA in 0.1 M PB for 1 h. The anterior sections, including the cornea and lens, were then excised to prepare eyecups. The retinal pigment epithelium (RPE)-choroid tissue was removed to create flat mounts, four equidistant incisions were made, and the retina was mounted between a microscope slide and coverslip. For samples to be sectioned and frozen, eyecups were soaked overnight in 30% sucrose in PBS and then embedded in Tissue-Tek (Miles Scientific, Naperville, IL) before preparing 5-μm-thick transverse retinal sections. These sections were incubated overnight at 4°C with primary Abs against mTOR (AF15371; R&D Systems), GFAP (12389; Cell Signaling Tech.), GS (MAB302; Millipore, Temecula, CA), CCL3 (Invitrogen), or CCR5 (Bioss, Woburn, MA). After three washes in PBST, they were incubated for 1 h with

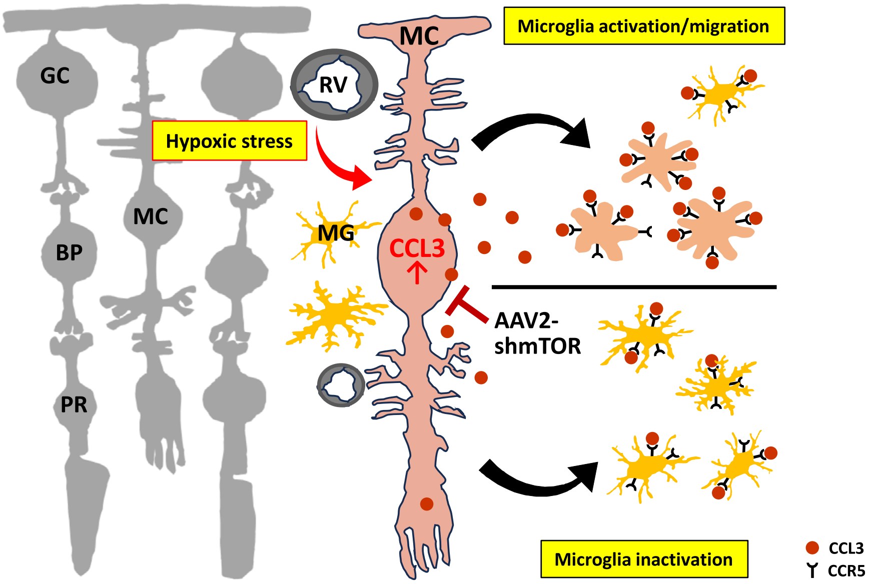

Figure 7. Schematic mechanism of anti-inflammatory effects by AAV2-shmTOR in the hypoxic retina

Administering the shmTOR virus effectively diminished mTOR signaling in activated Müller cells under hypoxic conditions. This reduced the release of the chemoattractant CCL3 from these cells, resulting in decreased mobilization of microglia and a reduction in inflammatory responses via the CCL3-CCR5 axis. GC, ganglion cell; BP, bipolar cell; PR, photoreceptor; RV, retinal blood vessel; MC, Müller cell.

secondary Abs Alexa Fluor 568 or 488 (Thermo Fisher Scientific) and stained with DAPI (D9542; Sigma-Aldrich). The samples were observed under an LSM 710 fluorescence confocal microscope (Carl Zeiss Microscopy, Jena, Germany). Images were captured using Zeiss Zen software (black edition, Carl Zeiss Microscopy) and analyzed with ImageJ (National Institutes of Health, Bethesda, MD). The images were quantified as a function of area at 200× magnification across 5 randomly chosen areas per sample and presented as a ratio relative to normal control mice.

Cytokine and chemokine immunoassay

The rMC1 cells were treated with a virus vector and subjected to hypoxia 24 h later. After 48 h, serum levels of VEGF-A, CXCL1, CXCL2, CXCL10, CCL2, CCL3, CCL5, and CCL7 were measured using a customized multiplex kit (ProcartaPlex multiplex immunoassay; Thermo Fisher Scientific). Each 96-well plate was combined with Ab magnetic beads, mixed for 2 min, and washed once. The standard and samples in 25 μL aliquots were added to the plate containing the magnetic bead mix, and 25 μL of universal assay buffer was added. The plate was incubated on a shaker at 500 rpm (Micromixer Mxi4t; Finepcr, Gunpo, Korea) at room temperature for 2 h in the dark. After incubation, the plate was washed three times with 150 μL of wash buffer using a microplate washer. A detector Ab was added to each well in 25 μL aliquots. The plate was then incubated on a shaker at 500 rpm at room temperature for 30 min in the dark. Finally, the plate was washed three times with 150 μL of wash buffer using a microplate washer. Streptavidin-PE was added to each well in 50 μL aliquots. The plate was incubated on a shaker at 500 rpm at room temperature for 30 min in the dark. After incubation, the plate was washed three times with wash buffer. Reading buffer was added to each well, and the plate was incubated on a shaker for 5 min to resuspend the beads before analysis. Finally, the plate was read on a Luminex CS1000 Autoplex Analyzer (PerkinElmer, Waltham, MA).

Statistical analysis

The results were statistically analyzed using Student's t test. The experiments were performed at least in triplicate. In the present experiments, we evaluated the statistical significance between the data from the MT or shCON group and the shmTOR group. Significant differences were determined at $^{*}p < 0.05$, $^{**}p < 0.01$, or $^{***}p < 0.001$. Graphs were used to visualize the data, including significance and mean ± SD values.

DATA AND CODE AVAILABILITY

The data used to support this study are available from the corresponding author upon reasonable request.

ACKNOWLEDGMENTS

This research was funded by CdmoGen Co., Ltd., a company known for its contributions to gene therapy and vaccine development. This work was supported by grants from the Basic Science Research Program through the National Research Foundation of Korea (2021R1A2C1093614 to H.L.).

AUTHOR CONTRIBUTIONS

H.L. and K.P. supervised the study; H.J.K. and J.-S.C. performed the *in vivo* experiments and analyzed the data; T.K.M., I.K.K., K.J.L., and J.H.K. conducted the *in vitro* experiments and analyzed the data; and T.K.M., I.K.K., A.R.H., and H.-N.W. designed the experiments and wrote the paper. H.L., K.P., and J.Y.L. reviewed and edited the manuscript. All authors have read and agreed to the published version of the manuscript.

DECLARATION OF INTERESTS

H.J.K., J.-S.C., and K.P. are CdmoGen Co., Ltd., employees, where K.P. has personal financial interests.

SUPPLEMENTAL INFORMATION

Supplemental information can be found online at https://doi.org/10.1016/j.omtm.2024.101404.

REFERENCES

1. Antonetti, D.A., Silva, P.S., and Stitt, A.W. (2021). Current understanding of the molecular and cellular pathology of diabetic retinopathy. Nat. Rev. Endocrinol. *17*, 195–206.
2. Deng, Y., Qiao, L., Du, M., Qu, C., Wan, L., Li, J., and Huang, L. (2022). Age-related macular degeneration: Epidemiology, genetics, pathophysiology, diagnosis, and targeted therapy. Genes Dis. *9*, 62–79.
3. Campochiaro, P.A. (2015). Molecular pathogenesis of retinal and choroidal vascular diseases. Prog. Retin. Eye Res. *49*, 67–81.
4. Uludag, G., Hassan, M., Matsumiya, W., Pham, B.H., Chea, S., Trong Tuong Than, N., Doan, H.L., Akhavanrezayat, A., Halim, M.S., Do, D.V., and Nguyen, Q.D. (2022). Efficacy and safety of intravitreal anti-VEGF therapy in diabetic retinopathy: what we have learned and what should we learn further? Expert Opin. Biol. Ther. *22*, 1275–1291.
5. Xu, M., Fan, R., Fan, X., Shao, Y., and Li, X. (2022). Progress and Challenges of Anti-VEGF Agents and Their Sustained-Release Strategies for Retinal Angiogenesis. Drug Des. Dev. Ther. *16*, 3241–3262.
6. Stewart, M.W. (2018). Extended Duration Vascular Endothelial Growth Factor Inhibition in the Eye: Failures, Successes, and Future Possibilities. Pharmaceutics *10*, 21.
7. Wallsh, J.O., and Gallemore, R.P. (2021). Anti-VEGF-Resistant Retinal Diseases: A Review of the Latest Treatment Options. Cells *10*, 1049.
8. Toutounchian, S., Ahmadbeigi, N., and Mansouri, V. (2022). Retinal and Choroidal Neovascularization Antivascular Endothelial Growth Factor Treatments: The Role of Gene Therapy. J. Ocul. Pharmacol. Therapeut. *38*, 529–548.
9. Ail, D., Malki, H., Zin, E.A., and Dalkara, D. (2023). Adeno-Associated Virus (AAV) - Based Gene Therapies for Retinal Diseases: Where are We? Appl. Clin. Genet. *16*, 111–130.
10. Wang, J.H., Gessler, D.J., Zhan, W., Gallagher, T.L., and Gao, G. (2024). Adeno-associated virus as a delivery vector for gene therapy of human diseases. Signal Transduct. Targeted Ther. *9*, 78.
11. Khanani, A.M., Thomas, M.J., Aziz, A.A., Weng, C.Y., Danzig, C.J., Yiu, G., Kiss, S., Waheed, N.K., and Kaiser, P.K. (2022). Review of gene therapies for age-related macular degeneration. Eye *36*, 303–311.
12. Yang, F., Zhang, H., Yu, X., Tao, Q., Zhao, C., An, J., Zhang, X., and Li, X. (2023). TNFAIP8 overexpression aggravates retinal pathophysiological features of diabetic retinopathy. Exp. Eye Res. *234*, 109572.
13. Campochiaro, P.A., Avery, R., Brown, D.M., Heier, J.S., Ho, A.C., Huddleston, S.M., Jaffe, G.J., Khanani, A.M., Pakola, S., Pieramici, D.J., et al. (2024). Gene therapy for neovascular age-related macular degeneration by subretinal delivery of RGX-314: a phase 1/2a dose-escalation study. Lancet *403*, 1563–1573.
14. Schnabolk, G., Parsons, N., Obert, E., Annamalai, B., Nasarre, C., Tomlinson, S., Lewin, A.S., and Rohrer, B. (2018). Delivery of CR2-fH Using AAV Vector Therapy as Treatment Strategy in the Mouse Model of Choroidal Neovascularization. Mol. Ther. Methods Clin. Dev. *9*, 1–11.
15. Ahn, J., Woo, H.N., Ko, A., Khim, M., Kim, C., Park, N.H., Song, H.Y., Kim, S.W., and Lee, H. (2012). Multispecies-compatible antitumor effects of a cross-species small-interfering RNA against mammalian target of rapamycin. Cell. Mol. Life Sci. *69*, 3147–3158.
16. Lee, S.H.S., Lee, J.Y., Choi, J.S., Kim, H.J., Kim, J., Cha, S., Lee, K.J., Woo, H.N., Park, K., and Lee, H. (2022). mTOR inhibition as a novel gene therapeutic strategy for diabetic retinopathy. PLoS One *17*, e0269951.
17. Lee, S.H.S., Chang, H., Kim, J.H., Kim, H.J., Choi, J.S., Chung, S., Woo, H.N., Lee, K.J., Park, K., Lee, J.Y., and Lee, H. (2020). Inhibition of mTOR via an AAV-Delivered shRNA Tested in a Rat OIR Model as a Potential Antiangiogenic Gene Therapy. Invest. Ophthalmol. Vis. Sci. *61*, 45.
18. Park, T.K., Lee, S.H., Choi, J.S., Nah, S.K., Kim, H.J., Park, H.Y., Lee, H., Lee, S.H.S., and Park, K. (2017). Adeno-Associated Viral Vector-Mediated mTOR Inhibition by Short Hairpin RNA Suppresses Laser-Induced Choroidal Neovascularization. Mol. Ther. Nucleic Acids *8*, 26–35.
19. Cha, S., Seo, W.I., Woo, H.N., Kim, H.J., Lee, S.H.S., Kim, J., Choi, J.S., Park, K., Lee, J.Y., Lee, B.J., and Lee, H. (2022). AAV expressing an mTOR-inhibiting siRNA exhibits therapeutic potential in retinal vascular disorders by preserving endothelial integrity. FEBS Open Bio *12*, 71–81.
20. Hammes, H.P. (2018). Diabetic retinopathy: hyperglycaemia, oxidative stress and beyond. Diabetologia *61*, 29–38.
21. Hommer, N., Kallab, M., Schlatter, A., Howorka, K., Werkmeister, R.M., Schmidl, D., Schmetterer, L., and Garhöfer, G. (2022). Retinal Oxygen Metabolism in Patients With Type 2 Diabetes and Different Stages of Diabetic Retinopathy. Diabetes *71*, 2677–2684.
22. Devoldere, J., Peynshaert, K., De Smedt, S.C., and Remaut, K. (2019). Muller cells as a target for retinal therapy. Drug Discov. Today *24*, 1483–1498.
23. Ou, K., Mertsch, S., Theodoropoulou, S., Wu, J., Liu, J., Copland, D.A., Scott, L.M., Dick, A.D., Schrader, S., and Liu, L. (2019). Muller Cells Stabilize Microvasculature through Hypoxic Preconditioning. Cell. Physiol. Biochem. *52*, 668–680.
24. Wang, M., and Wong, W.T. (2014). Microglia-Muller cell interactions in the retina. Adv. Exp. Med. Biol. *801*, 333–338.
25. Graca, A.B., Hippert, C., and Pearson, R.A. (2018). Muller Glia Reactivity and Development of Gliosis in Response to Pathological Conditions. Adv. Exp. Med. Biol. *1074*, 303–308.
26. Scott, A., and Fruttiger, M. (2010). Oxygen-induced retinopathy: a model for vascular pathology in the retina. Eye *24*, 416–421.
27. Maurer, M., and von Stebut, E. (2004). Macrophage inflammatory protein-1. Int. J. Biochem. Cell Biol. *36*, 1882–1886.
28. Schaller, T.H., Batich, K.A., Suryadevara, C.M., Desai, R., and Sampson, J.H. (2017). Chemokines as adjuvants for immunotherapy: implications for immune activation with CCL3. Expet Rev. Clin. Immunol. *13*, 1049–1060.
29. Wang, Y., Fung, N.S.K., Lam, W.C., and Lo, A.C.Y. (2022). mTOR Signalling Pathway: A Potential Therapeutic Target for Ocular Neurodegenerative Diseases. Antioxidants *11*, 1304.
30. Casciano, F., Zauli, E., Rimondi, E., Mura, M., Previati, M., Busin, M., and Zauli, G. (2022). The role of the mTOR pathway in diabetic retinopathy. Front. Med. *9*, 973856.
31. Sabatini, D.M. (2017). Twenty-five years of mTOR: Uncovering the link from nutrients to growth. Proc. Natl. Acad. Sci. USA *114*, 11818–11825.
32. Han, I.C., Cheng, J.L., Burnight, E.R., Ralston, C.L., Fick, J.L., Thomsen, G.J., Tovar, E.F., Russell, S.R., Sohn, E.H., Mullins, R.F., et al. (2020). Retinal Tropism and Transduction of Adeno-Associated Virus Varies by Serotype and Route of Delivery (Intravitreal, Subretinal, or Suprachoroidal) in Rats. Hum. Gene Ther. *31*, 1288–1299.
33. Srivastava, A. (2016). In vivo tissue-tropism of adeno-associated viral vectors. Curr. Opin. Virol. *21*, 75–80.
34. Russell, S., Bennett, J., Wellman, J.A., Chung, D.C., Yu, Z.F., Tillman, A., Wittes, J., Pappas, J., Elci, O., McCague, S., et al. (2017). Efficacy and safety of voretigene neparvovec (AAV2-hRPE65v2) in patients with RPE65-mediated inherited retinal dystrophy: a randomised, controlled, open-label, phase 3 trial. Lancet *390*, 849–860.
35. Ledford, H. (2017). FDA advisers back gene therapy for rare form of blindness. Nature *550*, 314.
36. Tang, Q., Gross, K.Y., Fakih, H.H., Jackson, S.O., Zain U I Abideen, M., Monopoli, K.R., Blanchard, C., Bouix-Peter, C., Portal, T., Harris, J.E., et al. (2024). Multispecies-targeting siRNAs for the modulation of JAK1 in the skin. Mol. Ther. Nucleic Acids *35*, 102117.
37. Bringmann, A., Pannicke, T., Grosche, J., Francke, M., Wiedemann, P., Skatchkov, S.N., Osborne, N.N., and Reichenbach, A. (2006). Muller cells in the healthy and diseased retina. Prog. Retin. Eye Res. *25*, 397–424.
38. Kobat, S.G., and Turgut, B. (2020). Importance of Muller Cells. Beyoglu Eye J. *5*, 59–63.
39. Eastlake, K., Banerjee, P.J., Angbohang, A., Charteris, D.G., Khaw, P.T., and Limb, G.A. (2016). Muller glia as an important source of cytokines and inflammatory factors present in the gliotic retina during proliferative vitreoretinopathy. Glia *64*, 495–506.
40. Qiu, A.W., Bian, Z., Mao, P.A., and Liu, Q.H. (2016). IL-17A exacerbates diabetic retinopathy by impairing Muller cell function via Act1 signaling. Exp. Mol. Med. *48*, e280.

41. Augustine, J., Pavlou, S., Harkin, K., Stitt, A.W., Xu, H., and Chen, M. (2023). IL-33 regulates Muller cell-mediated retinal inflammation and neurodegeneration in diabetic retinopathy. Dis. Model. Mech. *16*, dmm050174.

42. Zhang, H., Chen, K., Tan, Q., Shao, Q., Han, S., Zhang, C., Yi, C., Chu, X., Zhu, Y., Xu, Y., et al. (2021). Structural basis for chemokine recognition and receptor activation of chemokine receptor CCR5. Nat. Commun. *12*, 4151.

43. Skuljec, J., Sun, H., Pul, R., Bénardais, K., Ragancokova, D., Moharregh-Khiabani, D., Kotsiari, A., Trebst, C., and Stangel, M. (2011). CCL5 induces a pro-inflammatory profile in microglia *in vitro*. Cell. Immunol. *270*, 164–171.

44. Mafi, S., Mansoori, B., Taeb, S., Sadeghi, H., Abbasi, R., Cho, W.C., and Rostamzadeh, D. (2021). mTOR-Mediated Regulation of Immune Responses in Cancer and Tumor Microenvironment. Front. Immunol. *12*, 774103.

45. Okamoto, T., Ozawa, Y., Kamoshita, M., Osada, H., Toda, E., Kurihara, T., Nagai, N., Umezawa, K., and Tsubota, K. (2016). The Neuroprotective Effect of Rapamycin as a Modulator of the mTOR-NF-kappaB Axis during Retinal Inflammation. PLoS One *11*, e0146517.

46. Gu, L., Xu, H., Zhang, C., Yang, Q., Zhang, L., and Zhang, J. (2019). Time-dependent changes in hypoxia- and gliosis-related factors in experimental diabetic retinopathy. Eye *33*, 600–609.

47. Calderon, G.D., Juarez, O.H., Hernandez, G.E., Punzo, S.M., and De la Cruz, Z.D. (2017). Oxidative stress and diabetic retinopathy: development and treatment. Eye *31*, 1122–1130.

48. Shinojima, A., Lee, D., Tsubota, K., Negishi, K., and Kurihara, T. (2021). Retinal Diseases Regulated by Hypoxia-Basic and Clinical Perspectives: A Comprehensive Review. J. Clin. Med. *10*, 5496.

49. Babapoor-Farrokhran, S., Qin, Y., Flores-Bellver, M., Niu, Y., Bhutto, I.A., Aparicio-Domingo, S., Guo, C., Rodrigues, M., Domashevich, T., Deshpande, M., et al. (2023). Pathologic vs. protective roles of hypoxia-inducible factor 1 in RPE and photoreceptors in wet vs. dry age-related macular degeneration. Proc. Natl. Acad. Sci. USA *120*, e2302845120.

50. Lee, S.H.S., Chang, H., Kim, H.J., Choi, J.S., Kim, J., Kim, J.H., Woo, H.N., Nah, S.K., Jung, S.J., Lee, J.Y., et al. (2019). Effects of Stuffer DNA on the Suppression of Choroidal Neovascularization by a rAAV Expressing a mTOR-Inhibiting shRNA. Mol. Ther. Methods Clin. Dev. *14*, 171–179.

51. Smith, L.E., Wesolowski, E., McLellan, A., Kostyk, S.K., D'Amato, R., Sullivan, R., and D'Amore, P.A. (1994). Oxygen-induced retinopathy in the mouse. Invest. Ophthalmol. Vis. Sci. *35*, 101–111.

52. Connor, K.M., Krah, N.M., Dennison, R.J., Aderman, C.M., Chen, J., Guerin, K.I., Sapieha, P., Stahl, A., Willett, K.L., and Smith, L.E.H. (2009). Quantification of oxygen-induced retinopathy in the mouse: a model of vessel loss, vessel regrowth and pathological angiogenesis. Nat. Protoc. *4*, 1565–1573.

OMTM, Volume *33*

Supplemental information

mTOR downregulation promotes anti-inflammatory responses via the CCL3-CCR5 axis in hypoxic retinopathy

Tae Kwon Moon, Im Kyeung Kang, Kyoung Jin Lee, Ji Hyun Kim, Hee Jong Kim, A. Reum Han, Ha-Na Woo, Joo Yong Lee, Jun-Sub Choi, Keerang Park, and Heuiran Lee

Table S1. Summary of AAV2-shmTOR-SD toxicological study in NHP

A. Test Facility: Korea Institute of Toxicology, KRICT

B. Animal: Cynomolgus Monkey (*Macaca fascicularis*), 30 (Male 15, Female 15) animals

C. Test tissue list

Tissue Sampled	
Eyes (cornea, sclera, choroid and retina, lens, vitreous humo r, optic nerve, lacrimal gland/sac/duct)	Cerebrospinal fluid (CSF, ≥0.5 mL)
Brain (3 sections: frontal, mid brain, cerebellum)	Liver
Spleen	Lungs
Kidneys (cortex and medulla)	Heart
Ovaries	Testes
Uterus with cervix	Epididymides
Skeletal muscle	Colon
Salivary glands (all potential routes of viral clearance)	Lymph nodes (submandibular)
Urinary bladder	Thyroid gland

D. Quality control and detection range by PCR

The analytical method is considered to be suitable to analyze AAV2-shmTOR-SD in Cynomolgus Monkey within the range of 4×10^2 to 4×10^7 copies/500 ng.

E. Summary of analysis results

Toxicology	The toxicity was observed for 1 3 weeks after single intravitreal injection in NHP (Cynomolgus macaque).	No test article-related adverse effects were observ ed in any parameter after a single intravitreal injec tion of AAV2-shmTOR-SD at doses of 2.5 x 10^9 o r 1.25 x 10^{10} vg/eye. Based on the results, the No Observed Adverse Effect Level (NOAEL) was co nsidered to be 1.25 x 10^{10} vg/eye in both male and female under this study condition.
T-cell response	IFN-γ ELISpot assays and TNF -α measurement were tested usi ng the PBMC samples of NHP bloods taken in toxicology stud y.<br><br>The sampling of PBMC were p erformed in 3 time points after administration.	No immune activation against AAV capsid protein was observed following treatment of AAV2-shmT OR-SD.<br><br>Increasing of TNF-α was only observed on day 3 after intravitreal injection. This response was not o bserved at other time points (Day 22 and day 92) a fter administration of AAV2-shmTOR-SD.
Biodistribution	The biodistribution of AAV2-sh mTOR-SD was observed in eye and 17 organs (or tissues) for 1 3 weeks.<br><br>The viral vector was measured by real-time PCR analysis.	AAV2-shmTOR-SD was distributed primarily in t he vitreous humor and around injection site of the injected eye after a single intravitreal injection in c ynomolgus monkeys. AAV2-shmTOR-SD was bel ow quantifiable levels in non-ocular tissues.

Figure S1

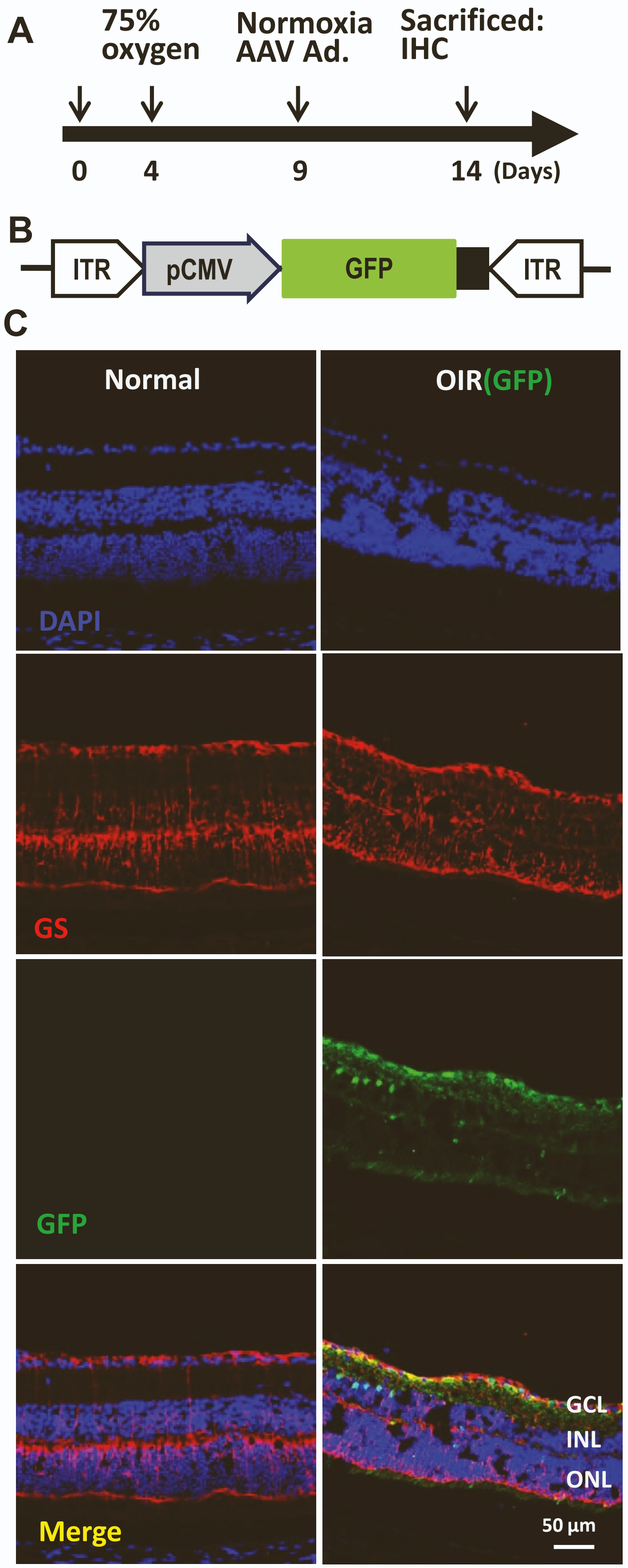

Figure S1. The transduction of **Müller cells by AAV2 in a rat OIR model.** (A) Diagram outlining the experimental setup. (B) Schematic overview of AAV2-GFP. (C) Immunohistochemistry targeting GFP and GS. The retina was analyzed after recovery to determine AAV2 tropism by immunohistochemically examining the GFP and GS expression pattern, demonstrating AAV's affinity for Müller cells. GCL, glial cell layer; INL, inner cell layer; ONL, outer cell layer.

Figure S2

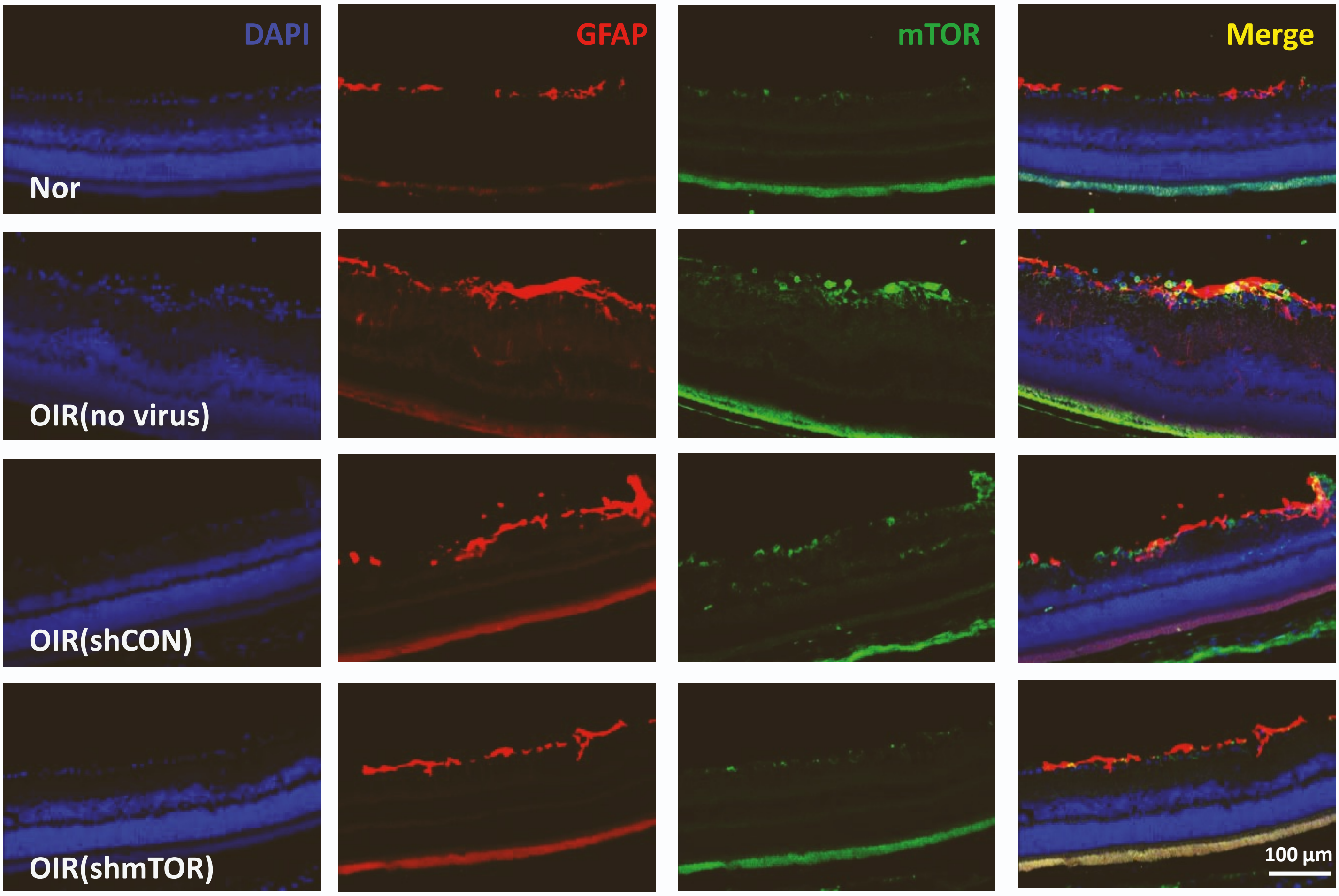

Figure S2. Immunohistochemical analysis targeting GFP and GFAP at low magnification of Fig. 1C.

Figure S3

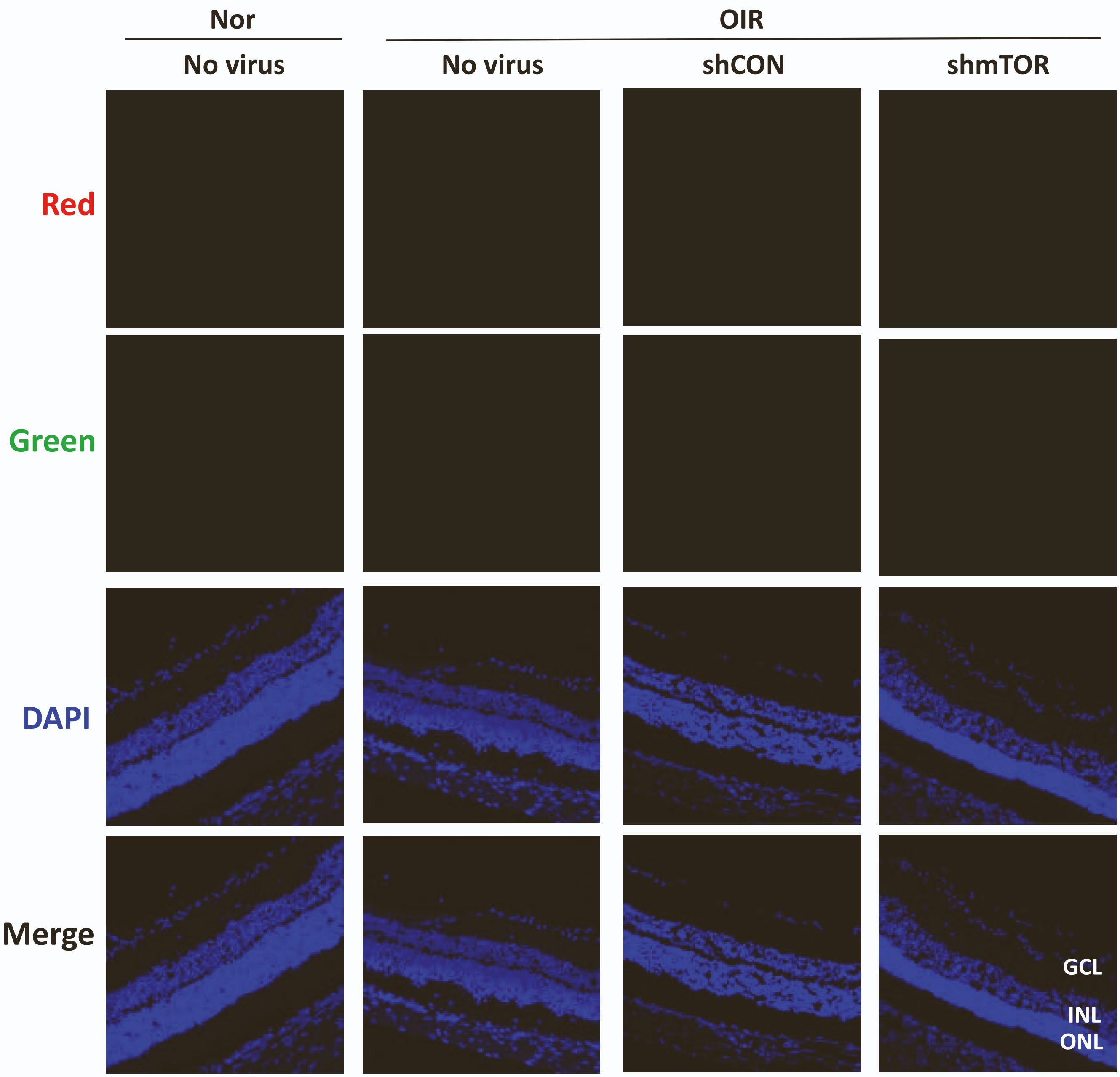

Figure S3. The immunohistochemical analysis was conducted without the use of a primary antibody, which served as a negative control of Fig. 1C.

Figure S4

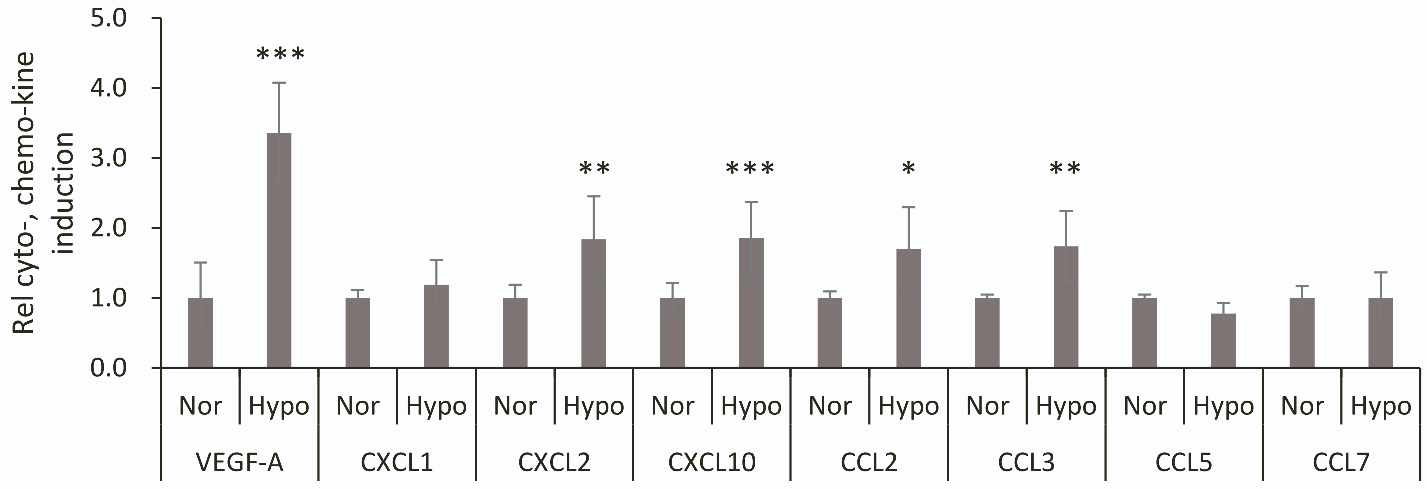

Figure S4. Relative secretion profiling of cytokines and chemokines from rMC1 cells under hypoxic conditions. Notably, the levels of CCL2, CCL3, CXCL2, CXCL10, and VEGF-A significantly increased under hypoxia. Results are presented as mean ± SD, with significant differences indicated (* $p < 0.05$, ** $p < 0.01$ and *** $p < 0.001$) based on three independent experiments. n≥7

Figure S5

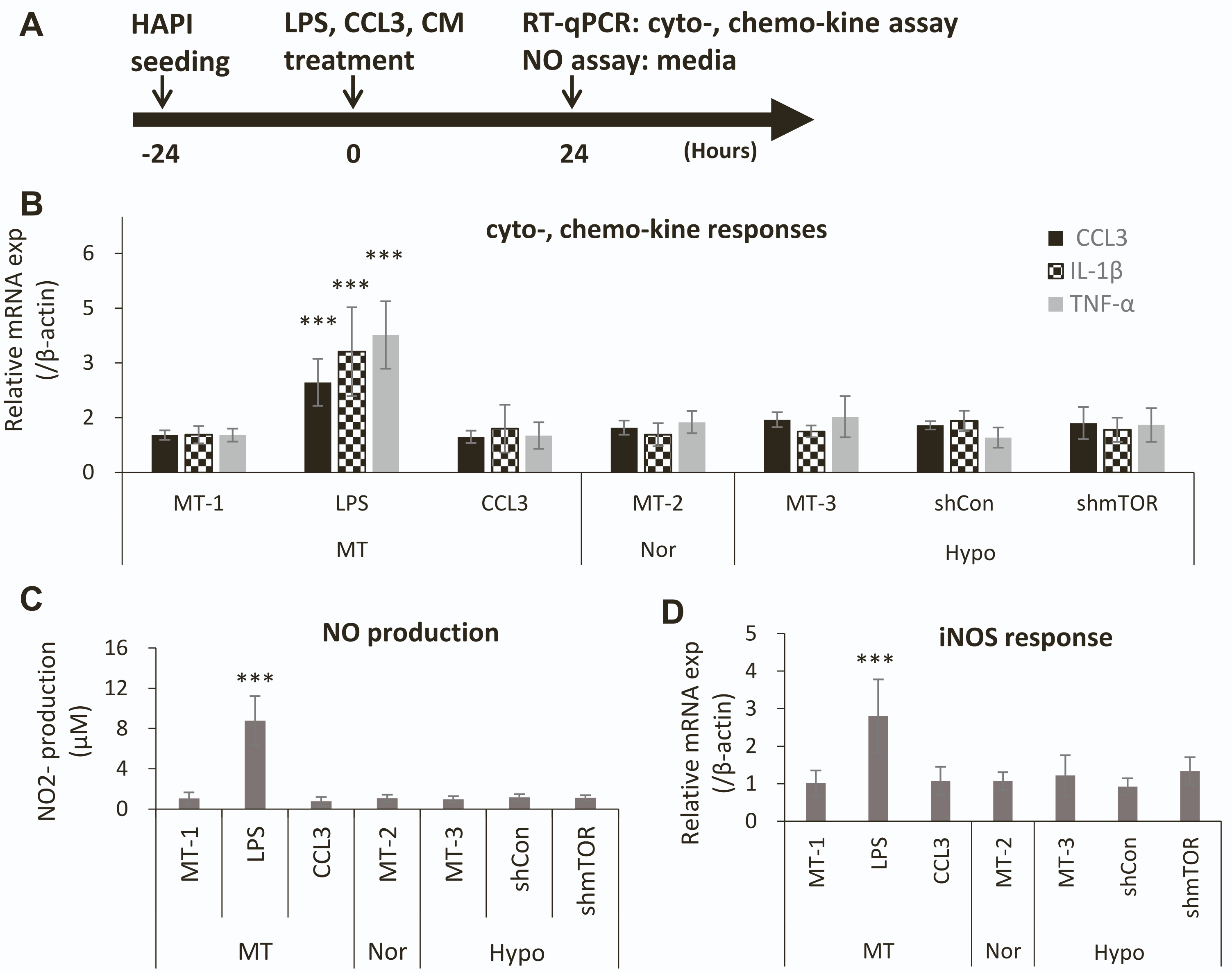

Figure S5. Responses of rat microglia cells following the exposure to CM from AAV2-shmTOR treated rMC1 cells under hypoxia. HAPI microglia cells were treated with LPS, CCL3, or CM. (A) Diagram outlining the experimental setup. (B) mRNA levels for CCL3, IL1-β, and TNF-α. (C) NO production. (D) iNOS mRNA level. Following the incubation with CM, there was no increase in the expression of various cytokines' mRNA or NO production. The results are presented as mean ± SD, with significant findings (*** $p < 0.001$) based on data from three experiments.

Supplemental Materials and Methods

Real-time quantitative polymerase chain reaction (RT-qPCR)

The total RNA preparation and RT-qPCR were conducted in accordance with the methodology described in the M & M. The primers used for CCL3, IL-1β, TNF-α and β-actin were as follows: CCL3, forward, 5'- AAGAGACCTGGGTCCAAGAA -3', reverse, 5'- GATTTGCAGGTGGCAGGAAT -3'; IL-1β, forward, 5'- GTTTGAGTCTGCACAGTTCC -3', reverse, 5'- AAGACACGGGTTCCATGGTG -3'; TNF-α, forward, 5'- TCGTAGCAAACCACCAAGC -3', reverse, 5'- TGCTGGTACCACCAGTTGG -3'; and β-actin, forward, 5'- TGAAGATCAAGATCATTGCTC -3', reverse, 5'- TGCTGGTACCACCAGTTGG -3'.

Tissue preparation and immunohistochemistry

The tissue preparation and immunohistochemistry were conducted according to the procedures described in the M & M.

Nitric oxide (NO) production

NO production was measured using the colorimetric Griess assay (Promega, Madison, WI) to assess nitrite concentration, a stable NO metabolite, in HAPI microglia cells. This was carried out after treatment with 1.0 μg/ml of LPS (Sigma-Aldrich), 100 ng/ml of CCL3 (Thermo Fisher Scientific), or conditioned media (CM) collected from rMC1 cells under various conditions. Absorbance readings were taken at 540 nm with a microplate reader.
